# Metal-Based PSMA Radioligands

**DOI:** 10.3390/molecules22040523

**Published:** 2017-03-24

**Authors:** Eleni Gourni, Gjermund Henriksen

**Affiliations:** 1 Institute of Basic Medical Sciences, University of Oslo, Oslo 0372, Norway; Gjermund.Henriksen@syklotronsenteret.no; 2 Norwegian Medical Cyclotron Centre Ltd., P.O. Box 4950 Nydalen, Oslo 0424, Norway; 3 Institute of Physics, University of Oslo, Oslo 0317, Norway

**Keywords:** prostate specific membrane antigen (PSMA), SPECT, PET, radionuclide therapy, intraoperative applications.

## Abstract

Prostate cancer is one of the most common malignancies for which great progress has been made in identifying appropriate molecular targets that would enable efficient in vivo targeting for imaging and therapy. The type II integral membrane protein, prostate specific membrane antigen (PSMA) is overexpressed on prostate cancer cells in proportion to the stage and grade of the tumor progression, especially in androgen-independent, advanced and metastatic disease, rendering it a promising diagnostic and/or therapeutic target. From the perspective of nuclear medicine, PSMA-based radioligands may significantly impact the management of patients who suffer from prostate cancer. For that purpose, chelating-based PSMA-specific ligands have been labeled with various diagnostic and/or therapeutic radiometals for single-photon-emission tomography (SPECT), positron-emission-tomography (PET), radionuclide targeted therapy as well as intraoperative applications. This review focuses on the development and further applications of metal-based PSMA radioligands.

## 1. Introduction

Prostate cancer is the most common malignancy found in men and the second leading cause of cancer death in the Western world. Even though prostate cancer is one of the few slow-growing cancers, it becomes potentially lethal upon metastasis. Consequently, early detection is particularly important for effective treatment. Functional imaging methods to detect prostate cancer include magnetic resonance imaging (MRI), computed tomography (CT), single-photon-emission tomography (SPECT) and positron emission tomography (PET). Prostate cancer is a complex and biologically heterogeneous disease and cannot be fully assessed with conventional imaging alone. MRI has shown high sensitivity for localizing prostate cancer, although it’s diagnostic performance and impact on the treatment regimen and varies in the patient populations studied so far. MRI is also not sensitive in detecting cancer in regions other than the peripheral zone of the prostate [1]. Both conventional imaging modalities, MRI and CT, focus on morphological changes, and their contribution to primary staging of prostate cancer is limited. Therefore, efforts to discover and evaluate new diagnostic and therapeutic biomarkers for prostate cancer continue. Radionuclide molecular imaging with SPECT or PET is poised to fill this unmet need through noninvasive detection of the multiple molecular and cellular processes that are active in prostate cancer patients [2]. Furthermore, the hybrid imaging techniques such as SPECT/CT and PET/CT combine functional and morphological information leading to high diagnostic accuracy. Nowadays, both hybrid imaging techniques are used in the clinical routine, not only as the primary staging tool in prostate cancer, but also in patients with suspected disease recurrence. The basis for the successful application of either SPECT or PET relies on the over-expression of specific receptors in various tumors [3,4]. In particular, prostate specific membrane antigen (PSMA) is a well-established enzyme/target for diagnostic and potential therapeutic applications via its targeting with suitable radiolabeled anti-PSMA tracers [5,6].

**Figure 1:**
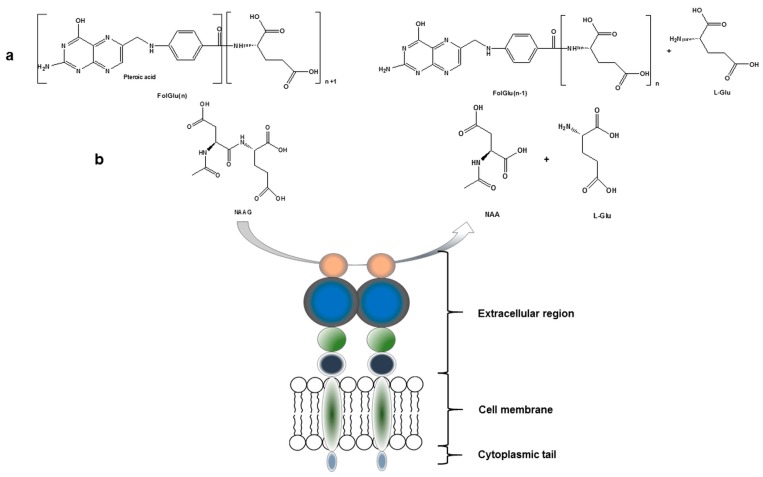
The enzyme actions of PSMA. (**a**) Glutamic acid is released from folate polyglutamate; (**b**) *N*-Acetyl-l-aspartyl-l-glutamate (NAAG) is hydrolyzed to aspartate (NAA) and glutamate (l-Glu).

PSMA, with a molecular weight of 100 kDa, consists of 750 amino acids and is a type II integral membrane glycoprotein. It has a unique structure containing three distinct parts; a 707-amino acid extracellular region, a cell membrane part of 24 amino acids and a cytoplasmic tail which contains 19 amino acids. PSMA is a key player in prostate carcinogenesis and disease progression, glutamatergic neurotransmission, and folate absorption [7]. These various functions and the tissue distribution of the protein result in different designations. The name which is also frequently used for this enzyme is glutamate carboxypeptidase II, or GCPII. Furthermore, in central nervous system, it metabolizes the brain neurotransmitter *N*-acetyl aspartyl glutamate (NAAG), and is named NAALADase. In the proximal small intestine, it removes γ-linked glutamates from poly-g-glutamated folate, which is reflected in its name, folate hydrolase FOLH1 (Figure 1) [5,7,8].

The NAALDase activity of PSMA has been extensively investigated for the development of PSMA-specific based ligands with the potential to be used for prostate cancer diagnosis and/or therapy. Briefly, the substrate, NAAG binds extracellularly to PSMA followed by hydrolysis of NAAG to glutamate and *N*-acetyl aspartate. Studies of the enzyme structure have shown the presence of a deep tunnel with a length of ~20 Å which connects the extracellular surface of PSMA with the active site of the enzyme, called the NAAG binding pocket. This binding pocket is also the binding site for the binding of the newly developed anti-PSMA ligands [9]. The proposed pharmacophore for PSMA active site can be divided into three parts: (i) three carboxylate groups; (ii) a carbonyl oxygen as part of the zinc complexation unit and (iii) nearby aromatic residues. More specifically, the crystal structure reveals two zinc atoms separated by approximately 3.2 Å. Each zinc atom in the PSMA active site is coordinated by three endogenous amino acids: a histidine (His-553 or -377), a terminal aspartate (Asp-453) or glutamate (Glu-425), and a bridged aspartate (Asp-387). The formed complex is finally surrounded by water molecules including conservative points [9,10]. Human PSMA has structural homology to another type II transmembrane glycoprotein, the transferrin receptor, which consists of 760 amino acids with a molecular weight of 190 kDa. Part of the extracellular domain of PSMA shows sequence similarity to the transferrin receptor (~54% similarity). The transferrin receptor however exists in dimeric form, whilst PSMA exists in dimeric/monomeric form although it is only enzymatically active as a dimer [11]. Both PSMA and transferrin receptor bind their ligands followed by internalization of the PSMA-bound ligand complex through the clathrin-coated pits [11,12,13]. PSMA and transferrin receptor are expressed on various tumor cells and inhibiting their functions may lead to implications for cancer therapeutics.

In pathological situations, PSMA is primarily expressed in the human prostate epithelium, salivary and lacrimal glands and kidneys with enhanced expression by almost all prostate cancers and further up-regulation in poorly differentiated, metastatic and hormone-refractory carcinomas without being shed into the circulation [5,14]. These characteristics render PSMA a promising target for imaging and therapy of prostate cancer. In particular, it is overexpressed by almost all prostate cancers with an increased expression by a factor of about 1000 in poorly differentiated, metastatic, and hormone-refractory cases [6,15,16]. Given the overexpression of PSMA in advanced prostate cancer, several efforts have been made especially during the last decade for the development of anti-PSMA-based imaging agents for SPECT and PET imaging.

## 2. PSMA-Based Radioligands

As part of the ongoing efforts of the scientific community to develop new anti-PSMA-specific ligands, various radiotracers have been reported, with some of them having shown great promise not only preclinically, but also in the clinical assessment. The dual nature of PSMA to act not only as a receptor protein, but also as an enzyme, has paved the way for the establishment of several approaches for its targeting via radiolabeled molecules. Firstly, based on the macromolecular protein structure of PSMA, specific monoclonal antibodies and smaller molecules called aptamers have been developed. These molecules bind tightly, selectively and specifically to PSMA [17,18]. Secondly, the enzymatic activity of PSMA served as the trigger for the synthesis and the further evaluation of a variety of anti-PSMA inhibitors of low molecular weight, with the potential to be used as nuclear imaging probes [19].

### 2.1. Anti-PSMA Monoclonal Antibodies as Radiotracers for Prostate Cancer Imaging and Therapy

PSMA was originally targeted by the monoclonal antibody 7E11-C5 (capromab) functionalized with the chelator diethylenetriaminepentaacetic acid (DTPA) and labeled with ^111^In to obtain ^111^In-7E11-C5 (ProstaScint^®^; Cytogen Corporation, Princeton, NJ, USA) [20,21,22,23]. ProstaScint^®^ binds to the intracellular site of PSMA (the amino terminus) which is accessible on necrotic tumors only [24,25]. Therefore, this tracer fails to gain wide acceptance in the field of nuclear medicine for the detection of prostate cancer. Hence, the need for the development of monoclonal antibodies which bind to the extracellular site of PSMA was evident. Consequently, several monoclonal antibodies, such as the humanized monoclonal antibody J591 [26,27] and three others murine monoclonal antibodies, named 3/A12, 3/E7 and 3/F11, were generated. These antibodies exhibit high and specific binding to cell-adherent PSMA [28,29].

Contrary to 7E11-C5, J591 can recognize PSMA that is present on the surface of nearly all prostate cancer tumors and circulating tumor cells. J591 is the first monoclonal antibody targeting the extracellular domain of PSMA as well as the first humanized monoclonal antibody to PSMA to be tested in humans. J591 has been evaluated preclinically for SPECT, PET as well as radioimmunotherapy. Its functionalization has been performed using a variety of chelators, such as 1,4,7,10-tetraazacyclododecane-1,4,7,10-tetraacetic acid (DOTA) and deferoxamine (DFO) and was further labeled with ^99m^Tc, ^111^In, ^89^Zn, ^64^Cu, ^177^Lu, ^90^Y [30,31,32,33,34,35,36,37,38,39,40,41]. PSMA SPECT imaging using ^99m^Tc-J591 depicts the presence of prostate cancer, and it can be also considered as a sensitive tool for the delineation of micro-invasion of the capsule, seminal vesicles or bladder neck. Although ^99m^Tc-J591 is of value in locating recurrence of the disease after radical prostatectomy, the results of the study indicated that extraprostatic cancer was not reliably detected. This difficulty may be due to the high activity of ^99m^Tc degradation products in the bladder [30]. ^111^In-J591 was also evaluated for SPECT imaging preclinicaly and in clinical trials. In the preclinical settings, the pharmacokinetics, biodistribution, and tumor uptake of ^111^In-J591 were compared with those of ^111^In-7E11 in nude mice with PSMA-positive LNCaP tumors [41]. The most important findings were that the blood clearance of ^111^In-J591 was relatively faster (8.98 ± 2.10, 4.78 ± 0.85, 2.52 ± 0.56 % IA/g at 2, 4 and 6 days p.i.) than that of ^111^In-7E11 (7.22 ± 0.46, 5.69 ± 1.00, 4.16 ± 0.41 % IA/g at 2, 4 and 6 days p.i.), leading to higher tumor-to-blood ratios (^111^In-J591: 1.62 ± 0.72, 3.35 ± 0.39, 6.07 ± 1.79, ^111^In-7E11: 1.34 ± 0.24, 2.83 ± 0.51, 4.57 ± 0.31 at 2, 4 and 6 days p.i. respectively). Furthermore, in clinical trials, ^111^In-J591 was found superior to ProstaScint^®^, revealing most soft-tissue and bone metastases [31,39,40]. J591 has also been radiolabeled with ^89^Zr, after its functionalization with 3.9 ± 0.3 accessible DFO chelators [34]. ^89^Zr-DFO-J591 was produced in high radiochemical yield (>77%) with radiochemical purity >99% and a specific activity of 181.7 ± 1.1 MBq/mg. The radiolabeled immunoconjugate remained active for up to 7 days at 37 °C. In vivo biodistribution studies revealed high uptake of ^89^Zr-DFO-J591 in LNCaP tumors after 24, 48, 96 and 144 h (34.4 ± 3.2 % IA/g; 38.0 ± 6.2 % IA/g; 40.4 ± 4.8 % IA/g; and 45.8 ± 3.2 % IA/g, respectively). The immunoPET imaging indicated that ^89^Zr-DFO-J591 shows high potential as a radiotracer for specific, non-invasive delineation of PSMA positive prostate cancers in vivo. In localized human prostate cancer cases, ^89^Zr-J591 bound to tumor foci in situ and PET identified primarily Gleason score 7 or greater and larger tumors. High grade tumors were generally better visualized with ^89^Zr-J591. These clinical data provide a basis on which to investigate the various roles of ^89^Zr-J591 PET in prostate cancer diagnosis, management and treatment [42]. The coupling of the chelator DOTA to J591 and the subsequent labeling with ^64^Cu led to ^64^Cu-J519, which was also evaluated for PET imaging preclinically [35]. ^89^Zr-labeled rather than ^64^Cu-labeled J591 was proved to successfully image PSMA-expression in preclinical prostate cancer models. J591 has also been adapted for radioimmunotherapy, and Ab–drug conjugates and therapeutic doses are well tolerated in patients. Multiple (two or three) administrations of ^177^Lu-J591 (30–60 mCi/m^2^) or ^90^Y-J591 (17.5 mCi/m^2^) over a 4- to 6-month period were tolerated by the patients with manageable thrombocytopenia. Furthermore, the clinical trials showed that even though a single large dose may deliver optimal radiation dose to kill a larger fraction of tumor cells, fractionated therapy offers the advantage of lower myelotoxicity and prolonged tumor response [33,36].

For the development of antibodies with a high cell binding activity which may have an important impact on antibody-based imaging and therapy, a series of monoclonal antibodies against PSMA, named 3/F11, 3/A12 and 3/E7, generated [28,29]. All three antibodies were able to bind efficiently to cell surface expressed PSMA. In addition, the in vivo behavior and tumor uptake of the ^64^Cu-labeled monoclonal antibodies 3/A12, 3/F11 and 3/E7 were also investigated. Radiolabeling of 3/A12, 3/F11 and 3/E7 with ^64^Cu through a conjugated DOTA moiety was accomplished without loss of immunoreactivity [43]. Biodistribution as well as PET imaging studies of the three ^64^Cu-labeled antibodies showed specific tumor uptake in PSMA-positive C4-2 tumors, whereas the uptake of tracers in PSMA-negative DU 145 tumors was found to be at the background level. In the same study, the pharmacokinetics of the ^64^Cu-DOTA-3/A12 Fab and ^64^Cu-DOTA-3/A12 F(ab′)2 fragments compared to that of ^64^Cu-DOTA-3/A12 were also evaluated at 3, 24 and 48 h after the injection of the radiotracers. Only a faint uptake of ^64^Cu-DOTA-3/A12 Fab and ^64^Cu-DOTA-3/A12 F(ab′)2 fragments in the PSMA-positive tumors was observed at all time points tested, indicating that the rate of clearance from blood was too fast to allow for sufficient tracer binding at the receptors. Furthermore, high kidney retention was also reported for both ^64^Cu-labeled fragments [43]. Behe et al. was published the labeling of 3/F11 through a conjugated DOTA moiety with ^177^Lu along with the in vitro and in vivo evaluation of ^177^Lu-DOTA-3/F11 [44]. The biodistribution data showed that the tumor uptake of ^177^Lu-DOTA-3/F11 gradually increased with time up to 72 h after the injection of the radiotracer. The high and persistent tumor uptake may be caused by a rapid internalization of the ^177^Lu DOTA-3/F11-PSMA complex into the target cells after binding [45], followed by metabolism and trapping of the radioactivity at the tumor site [46]. The same study also revealed that the treatment of C4-2 bearing prostate cancer xenografts with a single dose of 1 MBq ^177^Lu-DOTA-3/F11 led to a more than two-fold enhanced mean survival and a delay in tumor growth. However, this therapeutic window is small since mice treated with 2 MBq ^177^Lu-DOTA-3/F11 apparently died of myelotoxicity, which is the predominant dose-limiting factor in radioimmunotherapeutic approaches with ^177^Lu [36,47,48].

Despite the improved targeting of J591 and the subsequent generation of anti-PSMA monoclonal antibodies, the major impediment to the use of antibodies for diagnostic purposes continues to be the slow clearance from the non-target tissues. This, in most of the cases, is mitigated by introducing a time interval of several days between radiotracer administration and imaging. An alternative approach is to optimize the pharmacokinetics of the monoclonal antibodies by the generation of antibody fragments, such as single domain antibodies (V_HH_), called nanobodies. The comparatively low molecular mass of nanobodies leads both to a better permeability in tissues, and a shorter plasma half-life [49,50]. Recently, the anti-PSMA nanobodies, JVZ-007-c-myc-his and JVZ-007-cys, were developed and the nanobody-DTPA conjugates were labeled with ^111^In to obtain ^111^In-JVZ-007-c-myc-his and ^111^In-JVZ-007-cys [51]. ^111^In-JVZ-007-c-myc-his was evaluated in mice bearing PC-310 (PSMA+) and PC-3 (PSMA-) tumors, showing good and specific tumor targeting already at 4 h after injection, with low background intensity. Only kidney tracer uptake was high (110.89 ± 7.35 % IA/g at 4 h p.i.) which was reduced by about 75% after the administration of gelofusin and lysine [52,53]. The introduction of a cysteine at the C terminus for site-specific coupling to maleimide-DTPA and the labeling with ^111^In led to ^111^In-JVZ-007-cys. The in vivo evaluation of ^111^In-JVZ-007-cys in the same tumor model as described above revealed a further drop in renal uptake without loss of tumor targeting. These optimal characteristics of ^111^In-JVZ-007-cys may pave the way for its applicability to radionuclide therapy. The engineering of the new nanobody construct, based on JVZ-007, in which the c-myc-his tag was substituted with Cys tag, may have a considerable impact on the clinical implementation of a wide range of nanobodies in the field of nuclear medicine.

In addition to nanobodies, a variety of small, genetically engineered immunological constructs have been developed for a broader range of in vivo applications. Examples of these constructs include Fab (fragment antigen-binding), F(ab′)2, single-chain (sc) Fv, bis-scFV, diabodies and minibodies. As described above, their small size potentially gives them access to tissues that are poorly accessible by intact antibodies; rapid clearance from blood and non-targeted tissues, and at the same time they exhibit high affinity properties to antigens. As far the PSMA targeting concerns, an anti-PSMA minibody named IAB2M was genetically engineered from the parent antibody J591 and labeled with ^89^Zr [54]. Preclinical studies demonstrated faster clearance and rapid in vivo biokinetics, with efficient target penetration, allowing for high-contrast images within a few hours after the injection [46,55,56]. ^89^Zr-IAB2M [54] showed properties similar to that of ^89^Zr-J591 [34], coupled with a significantly faster blood clearance and lower uptake at 24 h after the injection in PSMA-positive tissue compared to ^89^Zr-J591, as shown by the PET images [57]. The latter finding can be attributed to the extended blood circulation of the intact antibody rather than loss of the affinity of IAB2M. Nevertheless, the longer circulation may benefit immunotherapies, but a key element for the successful diagnosis of cancer is a fast blood clearance in combination with high and retained tumor uptake, leading to high tumor-to-background ratios over time. The first-in-human imaging with ^89^Zr-IAB2M in metastatic prostate patients showed that PET imaging is feasible. Furthermore, favorable biodistribution profile in patients with advanced prostate cancer was also revealed. In addition to these characteristics, targeting of both bone and soft tissue lesions was observed after the administration of ^89^Zr-IAB2M [54].

Although immunoPET holds a very important role in the field of diagnostic imaging, the long blood circulation in combination with the nonspecific accumulation in normal tissues may lead to radiotoxicity of several vital organs such as the liver or the spleen. These obstacles could be overcome by generating smaller molecules which show fast blood clearance and retained radiotracer target specificity. The development of the genetically engineered immunological constructs properly modified in order to be used as nuclear probes can be considered as alternative imaging tools for cancer imaging.

### 2.2. Aptamers as Radiotracers for Prostate Cancer Imaging and Therapy

Aptamers are synthetic small molecules of 8–15 kDa is size which can be readily modified in a site-specific manner [58]. In particular, they are single-chain oligonucleotides that are selected from high-complexity RNA (or DNA) pools [58,59]. Their characteristics are described by tight binding to and inhibition of molecular targets. They can be chemically modified in such a way that they exhibit low immunogenicity and toxicity, in addition to an increased half-life in the circulation, rendering them attractive molecules either for diagnostic or therapeutic purposes. In fact, a variety of aptamer-based conjugates accumulate in tumors, suggesting that with specific modifications they may be used for imaging and potentially therapeutic applications [18,60,61,62]. Within this concept, two anti-PSMA aptamers, A9 and A10, have been developed and evaluated by Lupold et al. for their potential as specific targeting agents of prostate cancer [63]. They both exhibit specific binding to PSMA-positive cells in vitro. Additionally, A9g and A10-3.2 are the truncated versions of A9 and A10 respectively, which show enhanced binding affinity to PSMA and, importantly, enable inhibition of NAALADase/glutamate carboxypeptidase II activity. Rockey et al. published the functionalization of A10-3.2 with the chelators 1,4,7,10-tetraazacyclododecane-1,4,7-triaceticacid mono-*N*-hydroxy-succinimide (DOTA-NHS), *S*-2-(4-isothiocyanatobenzyl)-1,4,7-triazacyclononane-1,4,7-triacetic acid (*p*-SCN-Bn-NOTA), *p*-SCN-Bn-3,6,9,15-tetraazabicyclo [9.3.1]pentadeca-1(15),11,13-triene-3,6,9-triacetic acid (*p*-SCN-Bn-PCTA) and 1,8-diamino-3,6,10,13,16,19-hexaazabicyclo(6,6,6)eicosane (diAmSar) and their subsequent labeling with ^64^Cu [64]. The NOTA and PCTA constructs appear to be the most promising for further studies of the chelator-aptamer derivatives; however, in vivo images or biodistribution data of these ^64^Cu-labeled agents have so far not been reported.

### 2.3. PSMA Inhibitors of Low Molecular Weight

**Figure 2:**
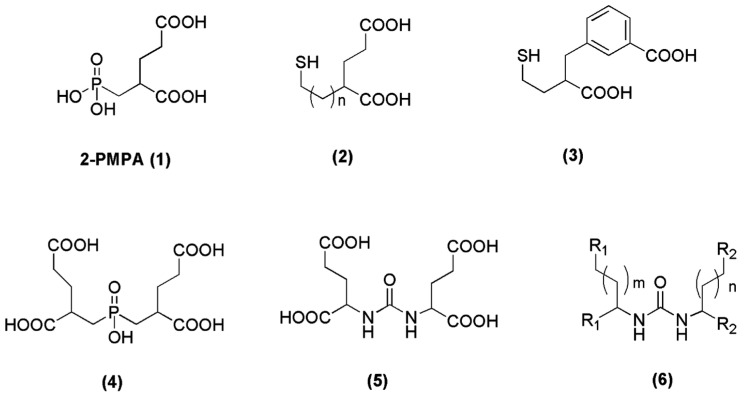
PSMA inhibitors. 2-PMPA (**1**) is the first potent PSMA inhibitor. The three groups of PSMA inhibitors include: thiols **2** and **3**, phosphonates **4** and ureas **5** and **6**.

Since 2-PMPA (compound **1**, Figure 2) was firstly reported as a potent PSMA inhibitor in 1996 [65], thorough efforts have been devoted to generating further molecules with inhibitory action towards PSMA. The main strategy for the discovery of those inhibitors was to find zinc-binding groups which are linked to a glutamate moiety. Three functionalities with affinity for zinc, including phosphonates (phosphates, phosphoramidates), thiols and ureas (compounds **2**–**6**, Figure 2) have been identified [66,67,68,69,70,71]. In the field of radiopharmaceutical development, a great number of phosphoramidates and urea inhibitors have been synthesized and modified accordingly in order to be labeled with a variety of radiometals. In the following chapters we will have a closer look on the derived radiotracers from these two categories.

## 3. Phosphoramidate-Metal Based PSMA Inhibitors

**Figure 3:**
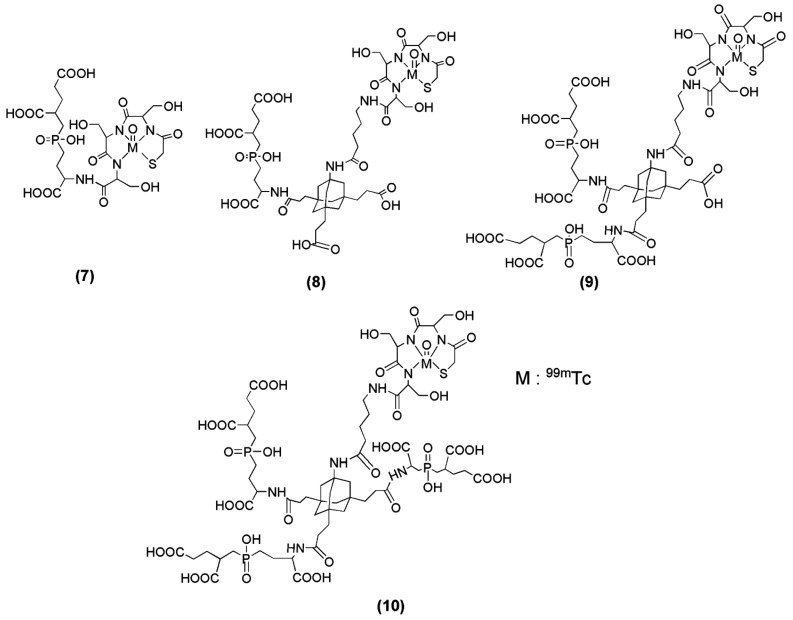
Chemical structures of the ^99m^Tc-labeled GPI (**7**) GPI monomer (**8**), GPI dimer (**9**) and GPI trimer (**10**) [72,73].

Misra et al. reported the development of a series of irreversible phosphoramidate inhibitors (Figure 3) that also target PSMA with high affinity and specificity [72]. The starting molecule for the synthesis of the new PSMA inhibitors was GPI (compound **7**, Figure 3). The phosphoramidate-based PSMA inhibitors were shown to rapidly internalize into subcellular organelles of PSMA-positive (PSMA+) prostate tumor cells, presumably through the enzyme-inhibitor complex [73]. ^99m^Tc-labeling of the derived inhibitors was obtained using a simple, cartridge-based, solid phase prelabeled 2-mercaptoacetyl-Ser-Ser-Ser- (MAS_3_) chelator. However, no in vivo imaging studies have been reported.

**Figure 4:**
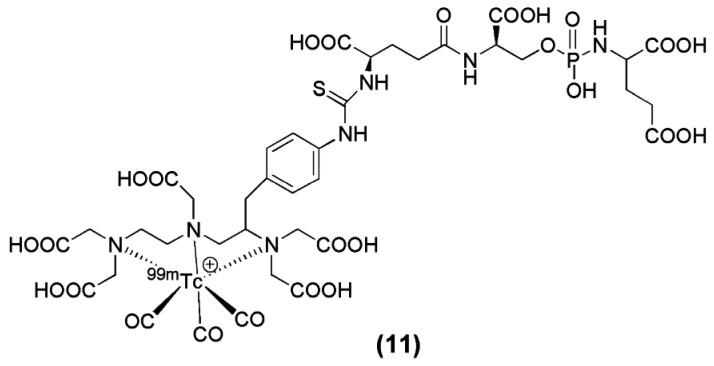
Chemical structure of ^99m^Tc(CO)_3_-DTPA-CTT-54 (**11**) [74].

The modification of another irreversible phosphoramidate inhibitor, named CTT-54 (compound **11**, Figure 4), for the delivery of ^99m^Tc(CO)_3_-DTPA-CTT-54, was reported by Nedrow-Byers et al. in 2012. ^99m^Tc(CO)_3_-DTPA-CTT-54 was evaluated in vitro and in vivo for its potential as a SPECT imaging nuclear probe for PSMA targeting [74]. Although DTPA may not be the ideal chelator for [^99m^Tc(CO)_3_]^+^, the results from the study justify a proof-of-concept evidence for the development of a next-generation of phosphoramidate-based PSMA inhibitors as SPECT imaging agents.

**Figure 5:**
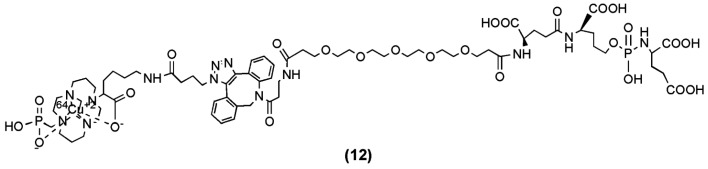
Chemical structure of ^64^Cu-ABN-1 (**12**) [75].

A PET tracer, ^64^Cu-ABN-1 (compound **12**, Figure 5) was generated from the irreversible phosphoramidate inhibitor of PSMA CTT-1297 after its conjugation to the phosphonate-pendant-armed cross-bridged chelator, 1,4,8,11-tetraazacyclotetradecane-1-(methanephosphonic acid)-8-(methanecarboxylic acid) (CB-TE1A1P) [75], and the incorporation of ^64^Cu [74]. ^64^Cu-ABN-1 was evaluated for its selective uptake both in vitro and in vivo with PSMA-positive cells. A particular focus was given to assess the ability of this ^64^Cu-labeled radiotracer to detect and distinguish elevated levels of PSMA in a panel of prostate tumor-bearing mouse models. The study showed that ^64^Cu-ABN-1 demonstrated selective uptake in PSMA-positive cells and tumors, which was also correlated to the level of PSMA expression. ^64^Cu-ABN-1 may serve as a non-invasive method to follow the progression of prostate cancer in the future.

The irreversible binding profile of the phosphoramidate-based PSMA inhibitors is desirable because it induces a higher rate of internalization compared to the slowly released, but reversiblebinding of phosphate-based inhibitors and the reversible profile urea- and phosphonic-based inhibitors. The phosphoramidate-based PSMA inhibitors are effective in delivering a therapeutically relevant dose from a relatively low dose due to the higher internalization rate which reduces radiation exposure of patients. The further optimization of this class of PSMA targeting agents has the potential to enable efficient in vivo targeting of PSMA positive tumors either for radiodiagnostic or radiotherapeutic applications.

## 4. Urea-Based PSMA Inhibitors

In 2001 the Kozikowski group was the first to first synthesize and evaluate urea-based PSMA inhibitors [70]. In 2002, the Pomper group at John Hopkins School of Medicine (Baltimore, MD, USA) published the synthesis and preclinical evaluation of the first radiolabeled urea-based PSMA inhibitor, (*N*-(*N*-[(*S*)-1,3-dicarboxypropyl]carbomoyl)-S-^11^C-methyl-l-cysteine) (^11^C-DCMC), also referred to as ^11^C-MeCys-C(O)-Glu (^11^C-MCG) [75]. To date, a variety of urea-based PSMA inhibitors have been developed and labeled with different SPECT (^99m^Tc, ^111^In), PET (^68^Ga, ^64^Cu), and therapeutic (^177^Lu) radiometals using several chelators and showed great promise not only preclinicaly but also in the clinical assessment. Apart from the radiometals to be used as the cytotoxic units, a considerable amount of urea-based PSMA inhibitors labeled with ^11^C, ^125^I, ^124^I, ^131^I and ^18^F, have also been reported [19,76,77,78,79,80,81,82,83,84,85,86,87,88,89,90,91,92,93,94,95,96]. These radiotracers were also able to successfully image PSMA-expressing xenografted mice, but since this is beyond the focus of this review no further information will be given here with regard to this topic.

The development of urea-based PSMA precursors for subsequent labeling with radiometals requires the attachment of a relatively bulky chelator to the peptidomimetic structure of the PSMA inhibitors. As it has been noticed earlier in this review, a ~20 Å deep tunnel connects the extracellular surface of PSMA with the active site of the enzyme, rendering difficult the entry of the radiometal-bearing chelator into the enzymatic binding site. Therefore, the insertion of a spacer between the urea-based motif and the chelator is essential. Although the binding affinity can be considered as the most crucial parameter which affects the tumor uptake, the overall pharmacokinetic performance of a radiotracer is determined by many other factors which certainly need to be taken into consideration during the development of potential radiopharmaceuticals. In particular, parameters such as the nature of the spacer, lipophilicity, charge, plasma protein binding and molecular weight also influence the pharmacokinetic performance of a radiotracer [97]. Indeed, the presence of the spacer not only greatly influences the affinity of the derived radiotracers towards PSMA but also the pharmacokinetic properties of the chelator-conjugated PSMA inhibitors [97].

### 4.1. ^99m^Tc-Labeled Urea-Based PSMA Inhibitors

**Figure 6:**
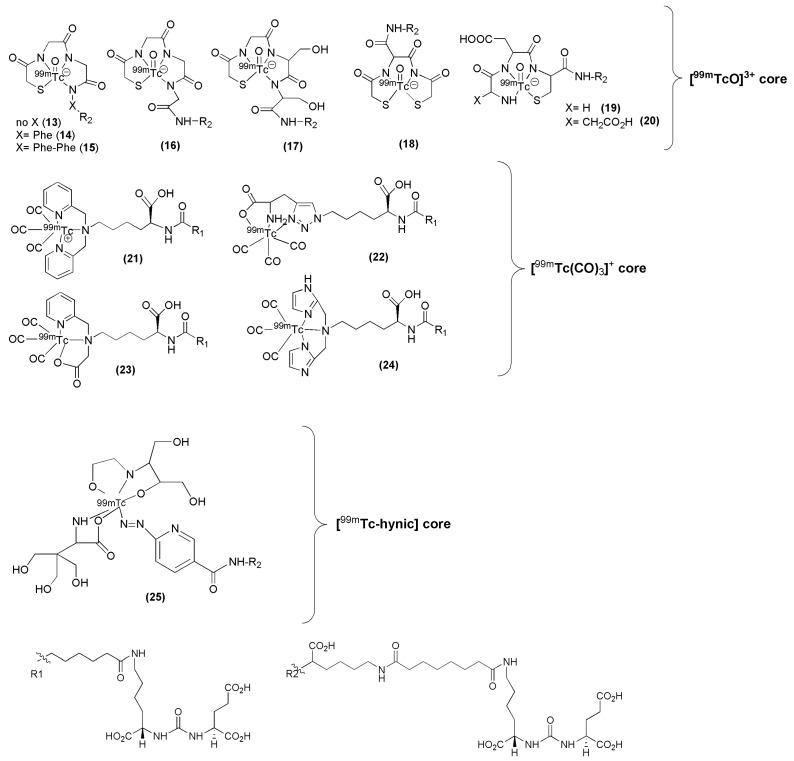
PSMA-inhibitors labeled via the ([^99m^TcO]^3+^) (**13**–**20**), ([^99m^Tc(CO)_3_]^+^) (**21**–**24**) and (^99m^Tc-hynic) (**25**) core [98,99].

^99m^Tc remains the radionuclide of choice for SPECT applications and a variety of PSMA inhibitors have been modified to facilitate efficient labeling with ^99m^Tc. Banerjee et al. have published a series of PSMA inhibitors suitable for labeling with ^99m^Tc [98,99]. The influence of the spacer length as well as the nature of different chelators was thoroughly investigated with respect to the overall performance of the derived ^99m^Tc-labeled Lys-urea-Glu-based PSMA inhibitors (compounds **13**–**25**, Figure 6, Table 1). In particular, the PSMA inhibitors are attached to hydrophilic ligands for radiolabeling with the ^99m^Tc(I)-tricarbonyl-labeled ([^99m^Tc(CO)_3_]^+^) core, traditional N_x_S_y_-based chelator for the ^99m^Tc-oxo ([^99m^TcO]^3+^) core, and a ^99m^Tc-organohydrazine (^99m^Tc-hynic) core. It was proved that parameters such the charge, polarity and hydrophilicity are crucial and contribute not only to the efficient targeting of PSMA by the ^99m^Tc-labeled agent but also to its pharmacokinetics.

The above tested low-molecular-weight, urea-based, ^99m^Tc-labeled, PSMA-targeted radiotracers, differ in the chelator and in the lipophilicity. Except for HYNIC-labeled ^99m^Tc-25 irrespective of charge and lipophilicity the radioligands ^99m^Tc-(13-14) enabled visualization of PSMA positive tumors (>20 % IA/g at 2 h p.i.) and kidneys. More specifically, the study showed that the degree of lipophilicity had a considerable effect on binding affinity as well as on in vivo performance. The ligands which showed higher lipophilic character revealed higher inhibitory activity, lower tumor and higher background uptake. Indeed, compounds **13** to **15**, which possess different degree of lipophilicity due to different number of Phe moieties (from zero (**13**) to three (**15**)) on the spacer, displayed considerable differences in tumor and background uptake as measured by SPECT/CT images from male SCID mice bearing PSMA+ PC3 PIP and PSMA− PC3 flu tumors at 2 h p.i. High gallbladder, liver, and gastrointestinal uptake were observed for ^99m^Tc-15 as compared to that of ^99m^Tc-13. Between the radioligands bearing the tricarbonyl core, ^99m^Tc-21 and ^99m^Tc-22, the neutral complex ^99m^Tc-22, exhibited favorable pharmacokinetics and promising SPECT/CT images, presumably because of the comparatively low background within the gastrointestinal tract relative to the positively charged ^99m^Tc-21 (^99m^Tc-21: small intestine: 1.29 ± 0.76 % IA/g, large intestine: 16.02 ± 12.39 % IA/g at 1 h p.i., ^99m^Tc-22: small intestine: 1.35 ± 0.19 % IA/g, large intestine: 0.29 ± 0.06 % IA/g at 1 h p.i.). In addition, ^99m^Tc-22, the most hydrophilic radioligand in the tricarbonyl series, had high, specific and retained tumor uptake in the PSMA+ PC3 PIP tumor (28.31 ± 4.38, 28.05 ± 2.04, 26.29 ± 7.45, 23.22 ± 6.02 % IA/g, at 0.5, 1, 2 and 5 h p.i. respectively). Another point which also needs to be pointed out is that the ^99m^Tc-oxo radioliagnds showed high and prolonged spleen uptake, which was not observed with the tricarbonyl-based radioligands. ^99m^Tc-22 proved superior with respect to tumor-to background ratios over time. Despite high tumor uptake, other compounds had either high gastrointestinal or spleen uptake, which cannot be easily attributed on their charge and lipophilicity. No clinical studies with of these ^99m^Tc compounds have been reported.

**Figure 7:**
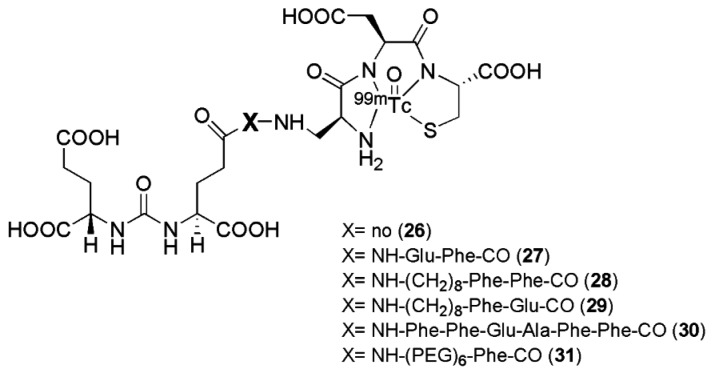
^99m^Tc-labeled PSMA inhibitors (**26**–**31**) [100].

Six targeted radioimaging agents labeled with ^99m^Tc derived from the Lys-urea-Glu peptidomimetic structure functionalized with the chelator Dap-Asp-Cys through spacers of different lengths (compounds **26**–**31**, Figure 7, Table 2) were investigated by Kularante et al. [100].

This study demonstrates the importance of the spacer between the urea-based PSMA inhibitors and the chelator which have been used for their functionalization. Docking studies revealed that coupling the urea analogue to an 8-aminooctanoic acid moiety (to avoid steric overlap within the narrow regions at the base of the tunnel) followed by two phenylalanine residues (for maximal interaction with hydrophobic pockets near the mouth of the tunnel) generated an agent (compound **28**) with high affinity and specificity. Also the most lipophilic compound was found to be the most affine towards PSMA. The in vivo data were in accordance with the findings from in vitro studies, with remarkable tumor uptake upon injection in nu/nu mice xenografted with LNCaP tumor cells (average 9.8 ± 2.4 % IA/g at 1 h p.i.) was observed for radiotracers **27**, **28**, and **29** with little accumulation in other tissues except the kidneys. These ligands have so far not been transferred to clinical evaluation.

**Figure 8:**
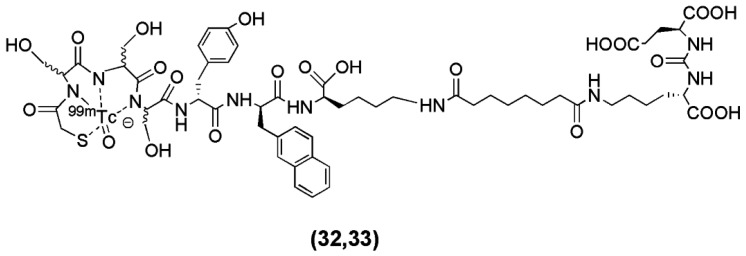
Chemical structure of ^99m^Tc-MAS_3_ (**32**)/mas_3_ (**33**)-y-nal-k-Sub-KuE (MAS_3_/mas_3_-D-Tyr-D-2-Nal-D-Lys-suberoyl-Lys-urea-Glu) [101].

Robu et al. recently published the synthesis of two ^99m^Tc-labeled anti-PSMA agents [101]. In this study the Lys-urea-Glu motif was coupled to the spacer d-Tyr-d-2-Nal-d-Lys-suberoyl (y-nal-k-Sub) to enhance interaction of the peptidic spacer with a remote arene binding site and the functionalization of the precursor took place through the conjugation of the chelators MAS_3_ (2-mercaptoacetyl-Ser-Ser-Ser-) (compound **32**, Figure 8) and mas_3_ (2-mercaptoacetyl-d-Ser-d-Ser-d-Ser-) (compound **33**, Figure 8). The derived conjugates labeled with ^99m^Tc (Table 3).

The main focus of the study was to investigate whether or not the all-L-amino acid chelator MAS_3_ is susceptible towards proteolytic degradation. As anticipated, no detectable influence of chelator stereochemistry on the outcome of the ^99m^Tc-labeling reaction was observed, since the formation of the ^99m^TcO-MAS_3_/mas_3_-complex should be independent from the spatial orientation of the serine side chains. On the other hand, the MAS_3_-chelating ^99m^Tc-labeled tracer showed substantially decreased in vivo stability compared to the mas_3_-derivative ^99m^Tc-PSMA-I&S (PSMA-I&S for Imaging and Surgery), for which only intact tracer was detected in blood, urine and kidney at 1 h p.i. Furthermore, ^99m^Tc-PSMA-I&S uptake in kidney and LNCaP-tumor was found to be high (186 ± 23, 8.28 ± 3.27 % IA/g at 1 h p.i., respectively) and specific, as shown by the blocking studies. The straightforward and reliable kit-production and the initial patient data indicate the potentiality of ^99m^Tc-PSMA-I&S as a SPECT imaging agent.

**Figure 9:**
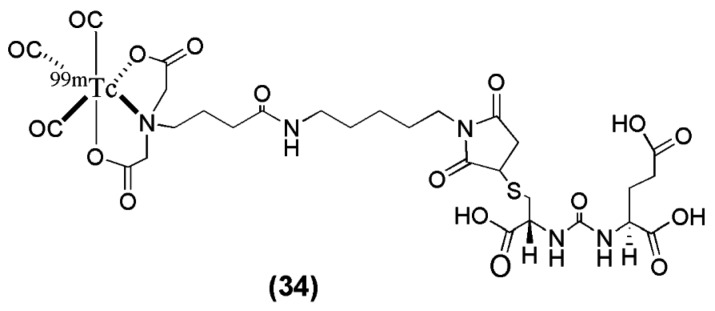
Chemical structure of ^99m^Tc-TMCE (**34**) [102].

In another study, Kimura et al. [102] synthesized and evaluated a novel anionic ^99m^Tc-tricarbonyl complex (^99m^Tc-TMCE) with high hydrophilicity due to strong polarity and electric charges with the intent to further decrease non-target tissue uptake and enhance renal clearance (compound **34**, Figure 9, Table 4). ^99m^Tc-TMCE was obtained in very low radiochemical yield (12–17%), introducing the implementation of a HPLC for purification of the radioligand. Compared to the neutral and positively charged tricarbonyl core-bearing radioligands published by Banerjee et al. the in vivo performance of the anionic ^99m^Tc-tricarbonyl complex ^99m^Tc-TMCE is characterized by a relatively long blood circulation and fast washout from the tumor (blood: 11.4 ± 2.3, 3.2 ± 0.5, 0.6 ± 0.6 ± 0.1 % IA/g and LNCaT-tumor: 4.0 ± 1.2, 12.8 ± 2.2 and 5.0 ± 2.7 % IA/g at 5, 30 and 120 min p.i. respectively) The outcome of the study demonstrated the effect of affinity, hydrophilicity, and electric charge in the pharmacokinetics of PSMA imaging probes. In particular, the hydrophilicity of both cationic and anionic charges led to rapid hepatobiliary clearance, although anionic charges might enhance renal clearance to a greater extent (kidney: 124.9 ± 26.2, 136 ± 6.4 and 56.8 ± 20.6 % IA/g at 5, 30 and 120 min p.i.). The implementation of this ^99m^Tc-labeled anti-PSMA radiotracer into the clinical pathway has not taken place yet.

**Figure 10:**
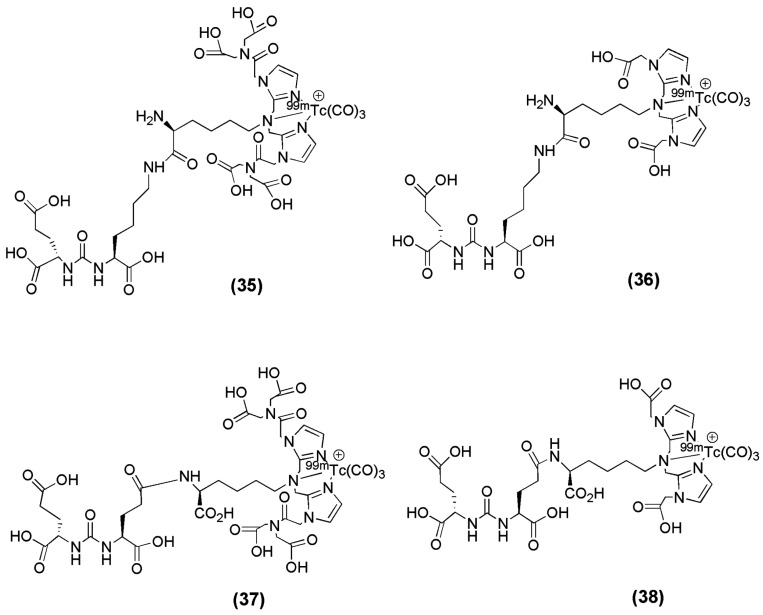
Chemical structures of ^99m^Tc-MIP-1428 (**35**), ^99m^Tc-MIP-1405 (**36**), ^99m^Tc-MIP-1404 (**37**) and ^99m^Tc-MIP-1427 (**38**) [103].

Four small-molecule inhibitors for PSMA labeled with ^99m^Tc via technetium tricarbonyl chemistry were reported by Hillier et al. [103]. The ^99m^Tc-labeled PSMA inhibitors derived from the glutamate-urea-glutamate or glutamate-urea-lysine pharmacophores after the incorporation of the chelators 2,2′-(2,2′-(azanediylbis(methylene))bis(1*H*-imidazole-2,1-diyl))diacetic acid (CIM) or 2,2′,2′′,2′′′-((2,2′-(2,2′-(azanediylbis(methylene))bis(1*H*-imidazole-2,1-diyl))bis(acetyl))bis(azanetriyl))tetraacetic acid (TIM) and the labeling with ^99m^Tc (compounds **35**–**38**, Figure 10) were evaluated in vitro and in vivo (Table 5).

The introduction of the chelators CIM and TIM to glutamate-urea-glutamate or glutamate-urea-lysine pharmacophores and the subsequent labeling with ^99m^Tc did not influence the affinity of the derived radioligands towards PSMA (Table 5), as their K_D_ values were found to be in the low nanomolar range. Furthermore, a side-by-side comparison of the affinity reveals a slightly higher affinity for PSMA of the glutamate-urea-glutamate-containing compounds, ^99m^Tc-MIP-1404 and ^99m^Tc-MIP-1427. The formal addition of an extra carboxylic acid group to the CIM chelator, yielding the TIM chelator, resulted in improved pharmacokinetics of the derived radioligands. Indeed, ^99m^Tc-MIP-1404 and ^99m^Tc-MIP-1428 displayed lower uptake in and more rapid clearance from blood, liver, kidneys (^99m^Tc-MIP-1404: blood: 0.13 ± 0.03 and 0.02 ± 0, liver: 0.14 ± 0.03 and 0.07 ± 0.01, kindey: 105 ± 37 and 12 ± 7 % IA/g at 1 and 4 h p.i. respectively, ^99m^Tc-MIP-1428: blood: 0.44 ± 0.07 and 0.14 ± 0.03, liver: 0.27 ± 0.05 and 0.14 ± 0.04, kidney: 136 ± 6 and 63 ± 27 % IA/g at 1 and 4 h p.i. respectively), and most other nontarget tissues than did ^99m^Tc-MIP-1405 and ^99m^Tc-MIP-1427 (^99m^Tc-MIP-1405: blood: 0.72 ± 0.19 and 0.26 ± 0.03, liver: 0.43 ± 0.14 and 0.19 ± 0.05, kidney: 157 ± 69 and 14 ± 6 % IA/g at 1 and 4 h p.i. respectively, ^99m^Tc-MIP-1427: blood: 0.55 ± 0.08 and 0.28 ± 0.03, liver: 0.83 ± 0.24 and 0.76 ± 0.26, kidney: 149 ± 24 and 142 ± 39 % IA/g at 1 and 4 h p.i. respectively). The chelator TIM did not diminish uptake or retention in the LNCaP xenograft tumor, as there was no statistical difference between the tumor uptake of any of the compounds at either the 1 h or the 4 h time points. More specifically, uptake in LNCaP xenografts ranged from 9.3% to 12.4% injected dose per gram at 1 h after injection and from 7.2% to 11.0% at 4 h, with tumor-to-blood ratios ranging from 29:1 to 550:1 and tumor–to–muscle ratios ranging from 31:1 to 157:1 at 4 h. ^99m^Tc-MIP-1404 is currently under clinical investigation in a phase II trial.

### 4.2. ^68^Ga-Labeled Urea-Based PSMA Inhibitors

Because of its availability from the ^68^Ge/^68^Ga generator systems and the relative ease of labeling chemistry, the positron emitter ^68^Ga (t_½_ = 68 min, β^+^_max_ = 1899 keV) has gained increasing interest in the field of molecular imaging [104] and can considered a PET-applicable counterpart of ^99m^Tc.

**Figure 11:**
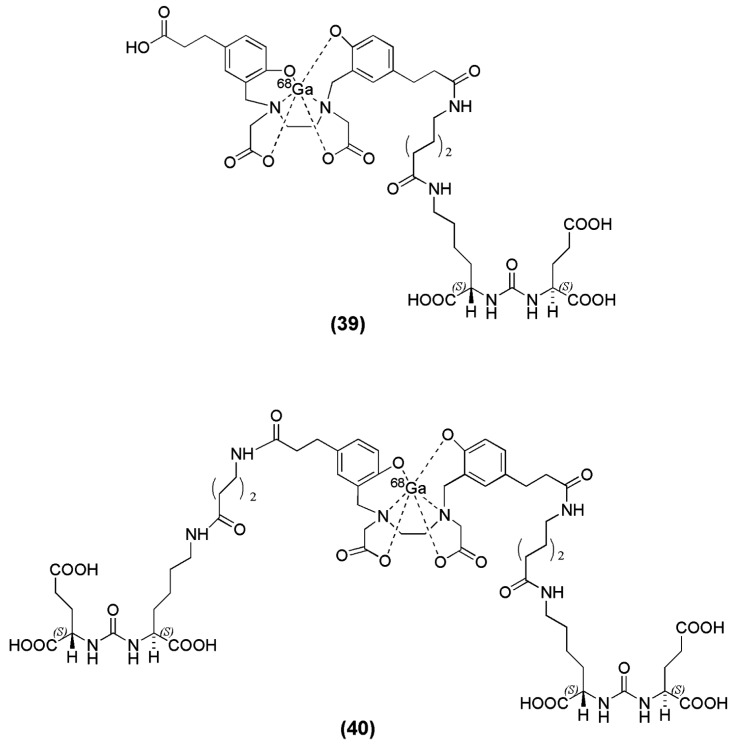
Chemical structures of the monomer ^68^Ga-PSMA-11 (**39**) and the dimer ^68^Ga-PSMA-10 (**40**) [106,130].

Many efforts have been made with respect to the development of ^68^Ga-PSMA-based imaging agents in the hope of developing a cyclotron-independent nuclear probe for prostate cancer imaging. A variety of PSMA-based inhibitors have been generated where several chelators suitable for labeling with ^68^Ga as well as spacers have been investigated. In solution, gallium is most stable in the +3-oxidation state and forms stable complexes with several chelating agents. The HBED chelator (*N*,*N*′-bis[2-hydroxybenzyl] ethylenediamine-*N*,*N*′-diacetic acid) which contains an amine-phenol backbone which renders it suitable for complexation with +3 metal ions, and it forms a thermodynamically stable complex with ^68^Ga even at room temperature [105]. A representative example of PSMA-inhibitors associated with the chelator HBED, is the peptidomimetic structure Lys-urea-NH-Glu, published by Eder et al., when coupled to the spacer 6-aminohexanoic acid (Ahx) and functionalized with the chelator *N*,*N*′-dis[2-hydroxy-5-(carboxyethyl)benzyl] ethylenediamine-*N*,*N*′-diacetic acid (HBED-CC) to obtain HBED-CC-Ahx-Lys-urea-Glu (HBED-CC-PSMA or PSMA-11) (compound **39**, Figure 11) [106]. HBED-CC has a dual nature, it also acts as a lipophilic moiety due to the presence of the two phenolic rings and therefore an aliphatic spacer such as Aca is adequate for providing the required lipophilicity and retaining the affinity of the derived ligand towards PSMA (K_i_ = 12.1 ± 2.1 nM). Radiolabeling with ^68^Ga was performed at pH 4.2 by incubating PSMA-11 in a mixture consisting of 50–100 MBq [^68^Ga]Ga^3+^ and HEPES. An amount of 0.1 nmol of PSMA-11 at a final concentration of 1.7 μM led to a radiochemical yield of more than 99% in less than 1 min at room temperature, with specific activities in the range og 500–1000 GBq/μmol. These are the highest specific activities which have been reported for ^68^Ga-lebeled PSMA inhibitors. In order to achieve comparable high radiochemical yields with the DOTA-conjugated PSMA inhibitors, the compounds were incubated for 2 min at 80 °C using 1 nmol of precursor [106]. ^68^Ga-PSMA-11 exhibited fast blood clearance, relatively low liver uptake (0.87 ± 0.05 % IA/g at 1 h p.i.), and high specific uptake in PSMA-expressing tissues and tumor (tumor uptake 7.7 ± 1.5 % IA/g at 1 h p.i.). The ability to image PSMA using ^68^Ga-HBED-CC-PSMA showed great promise not only preclinically but also clinically [106,107,108,109,110,111,112,113,114,115,116,117,118,119,120,121,122,123,124,125,126,127,128,129]. The dimerization of the same pharmacophore, Lys-Urea-Glu, coupled to the same spacer Ahx, through the chelator HBED-CC (PSMA-10) (compound **40**, Figure 11) was also published by Schäfer et al. [130]. The dimer was also labeled with ^68^Ga to obtain ^68^Ga-PSMA-10 and evaluated in vitro and in vivo compared to ^68^Ga-PSMA-11. Despite the fact that the preclinical evaluation revealed that ^68^Ga-PSMA-10 was superior to ^68^Ga-PSMA-11 in terms of binding affinity and tumor to background ratios (IC_50_ = 3.9 ± 1.8 nM with a target to non-target ratio of 26.5 at 1 h p.i. as compared to a value of 9.2 for the monomer at the same time point), most of the clinical studies, so far, have been conducted using ^68^Ga-PSMA-11.

**Figure 12:**
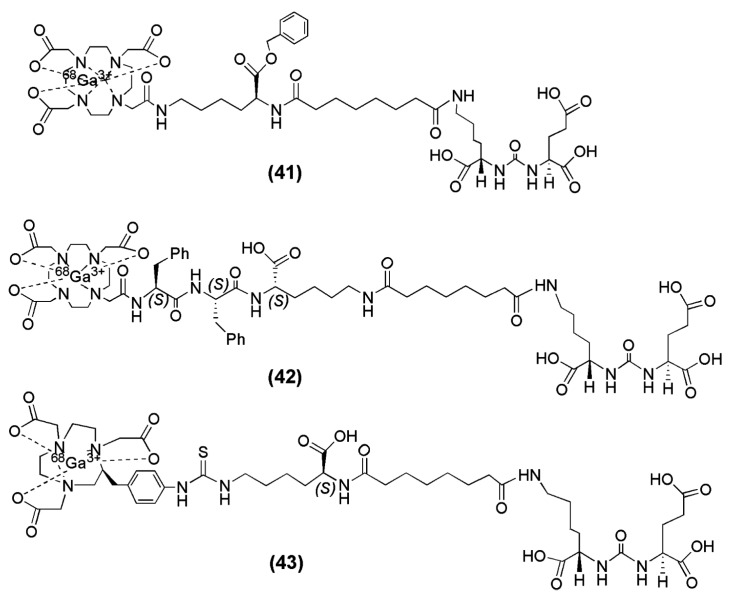
Chemical structures of the two DOTA- (**41** and **42**) and the NOTA-conjugated (**43**) PSMA inhibitors labeled with ^68^Ga [131,132].

**Figure 13:**
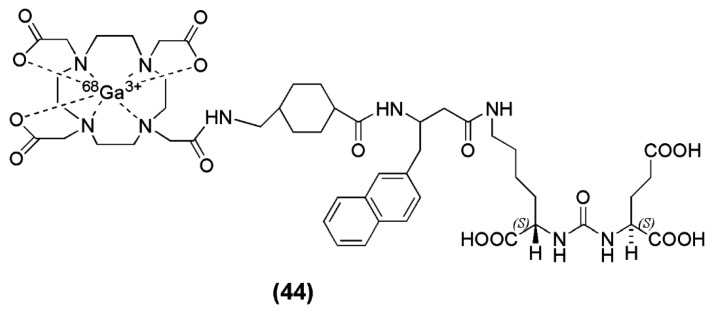
Chemical structure of ^68^Ga-PSMA-617 (**44**) [133].

In the field of radiopharmaceutical chemistry, the cyclic chelator 1,4,7,10-tetraazacyclododecane-1,4,7,10-triacetic acid (DOTA) has been used for complexation of +3 radiometal ions such as ^68^Ga, ^90^Y and ^177^Lu. Banerjee et al. described two DOTA-conjugated PSMA inhibitors based on the Lys-Urea-Glu construct that confers PSMA specificity (compounds **41** and **42**, Figure 12) and are suitable for labeling with ^68^Ga [131]. The difference between those two tracers is confined to the spacer unit. More specifically, a benzyl group was inserted into compound **41** (Figure 12) in order to provide a chromophore that facilitates purification. On the other hand, two phenylalanines were added to compound **42** (Figure 12) in order to offset its high hydrophilicity and enable sustained retention and potentially higher absolute uptake in tumor [98]. Furthermore, in a recent study from the same group [132], a new radiotracer containing the macrocyclic chelator NOTA was prepared, since NOTA has been shown to be an effective chelator for ^68^Ga (compound **43**, Figure 13,). A head-to-head preclinical comparison of ^68^Ga-42 with ^68^Ga-43 (Figure 12) was conducted. ^68^Ga-PSMA-11, the radioligand which has been used throughout Europe in clinical trials, was also included in the study for comparison ^68^Ga-41, ^68^Ga-42 and ^68^Ga-43 were directly labeled with the eluted ^68^Ga from the ^68^Ge/^68^Ga generator without the use of a buffer solution within 10 min at 90–95 °C. The yields ranged from 60% to 70% with HPLC purification and the reported radiochemical purities were >99% with specific activities >168 GBq/μmol. Both ^68^Ga-41 (−3.9) and ^68^Ga-43 (−4.01 ± 0.16) were found to be 1 order of magnitude more hydrophilic than ^68^Ga-42 (−3.0 ± 0.1). It is evident that ^68^Ga-42 is more lipophilic than ^68^Ga-41 and ^68^Ga-43, which is reasonable because of the presence of two phenylalanine residues in the spacer. The corresponding metal-labeled compounds demonstrated high binding affinity to PSMA, with Ki values ranging from 0.33 to 29 nM (compound **41** was the less affine towards PSMA).

Both compounds, ^68^Ga-41 and ^68^Ga-42, exhibited PSMA specific tumor imaging in vivo. Nevertheless, the improved tumor-to-background ratios of ^68^Ga-42 at later time points after injection renders it a more promising candidate for clinical translation. The PSMA+ PC3 PIP tumors as well as PSMA positive organs such as, kidney and urinary bladder were clearly delineated already at 15 min p.i. More specifically, the NOTA-conjugated radioligand ^68^Ga-43 exhibited the highest tumor uptake with 42.2 ± 6.7 % IA/g at 1 h p.i. and the fastest background clearance. The PSMA+ PC3 PIP-to-PSMA− PC3 flu tumor ratios were 110 ± 22 at 1 h, 232 ± 26 at 2 h, and 182 ± 15 at 3 h p.i. Renal uptake for ^68^Ga-43 was highest at 1 h, 106 ± 23 % IA/g, much higher than that seen for the DOTA-conjugated radioligand ^68^Ga-42 (26.5 ± 6.9 % IA/g), and showed faster renal clearance, which decreased to 34.7 ± 5.7 % IA/g by 2 h p.i. There was no significant difference in PSMA+ PIP tumor uptake between ^68^Ga-42 and ^68^Ga-PSMA-11 or between ^68^Ga-43 and ^68^Ga-PSMA-11 (*p* > 0.05) at any time-point. ^68^Ga-PSMA-11 demonstrated the highest and retained uptake in normal tissues, including kidney, blood, spleen, salivary glands and PSMA-negative PC3 flu tumors up to 3 h post-injection. This preclinical evaluation showed that ^68^Ga-43 was superior for PSMA-targeted PET imaging in clinical settings.

Benešová et al. [133], published the synthesis and preclinical evaluation a novel theranostic compound termed PSMA-617 (compound **44**, Figure 13). In this case, the chelator DOTA, was conjugated to the pharmacophore Lys-urea-Glu via a naphthylic spacer. PSMA-617 was labeled with [^68^Ga]Ga^3+^ eluate in HEPES buffer, pH 4.0, within 15 min at 95 °C, with a radiochemical yield of more than 90% and a specific activity in the range of 14–140 GBq/μmol. The study showed that the presence of the naphthylic linker has a significant impact on the tumor-targeting as well as on the pharmacokinetics and the resulting imaging contrast. In fact, ^68^Ga-PSMA-617 (K_i_ = 6.40 ± 1.02 nM) was superior to ^68^Ga-PSMA-11 (K_i_ = 12.1 ± 2.1 nM) as far as the affinity towards PSMA and efficacy of internalization (up to 17.67 6 4.34 percentage injected activity/10^6^ LNCaP cells) into the cancer cells concerns. Biodistibution studies upon injection of ^68^Ga-PSMA-617 on LNCaP tumor bearing mice 1 h p.i. revealed a high specific uptake in LNCaP tumors (8.47 ± 4.09 % IA/g; 0.98 ± 0.32 % IA/g by coinjection of 2-PMPA) and in the kidneys (113.3 ± 24.4 % IA/g). Other organs such as the liver (1.17 ± 0.10 % IA/g), lung (1.41 ± 0.41 % IA/g), and spleen (2.13 ± 0.16 % IA/g) showed rather low uptake. Furthermore, ^68^Ga-PSMA-617 dynamic PET imaging showed that the maximum kidney uptake was reached within 15 min after injection and decreased substantially as early as 20 min p.i. High and sustained tumor uptake was observed. The fast kidney clearance emboldened clinical translation of this compound.

**Figure 14:**
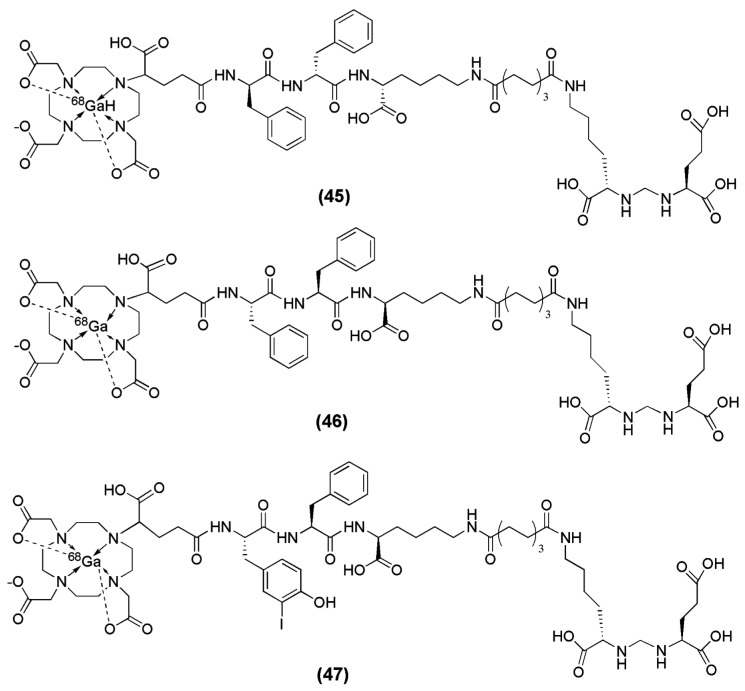
Chemical structures of the three generations (DOTAGA-FFK(Sub-KuE) (**45**), DOTAGA-ffk(Sub-KuE) (**46**) and of DOTAGA-(I-y)fk(Sub-KuE) (**47**)) PSMA inhibitors labeled with ^68^Ga [134,135].

When Lys-Urea-Glu (KuE) was coupled to the spacer Phe-Phe-Lysine-suberoyl (l-amino acid spacer, FFK-Sub) and functionalized with the chelator (1-(1,3-carboxypropyl)-4,7,10(carboxymethyl)-1,4,7,10 tetraazacyclododecane (DOTAGA) DOTAGA-FFK(Sub-KuE) was obtained [134]. HEPES buffer was used for the labeling with ^68^Ga (pH 4.5). The reaction completed within 5 min at 95 °C and resulted in a radioligand with a specific activity of 250–300 GBq/μmol. However, a rapid in vivo metabolysis of the ^68^Ga-labeled radiovector was demonstrated. In the same report by Weineisen et al. the inclusion of a D-amino acids spacer led to an in vivo metabolic stable radiotracer (DOTAGA-ffk(Sub-KuE)) (compounds **45**–**47**, Figure 14).

In an attempt from the same group to further optimize this second-generation of the PSMA inhibitor with respect to its affinity towards the PSMA enzyme, additional modifications on the spacer unit were undertaken. [135]. For that purpose, d-Phe (f) was substituted with d-I-Tyr (I-y) and DOTAGA-(I-y)fk(Sub-KuE) was synthesized (Figure 14). DOTAGA-(I-y)fk(Sub-KuE) is also termed PSMA I&T (PSMA I&T for Imaging &Therapy), since it can be labeled with both the diagnostic radionuclide ^68^Ga and the therapeutic radionuclide ^177^Lu. The necessary modifications for the generation of a metabolically stable radioligand did not alter the affinity of the derived ligands towards PSMA, since all the precursors as well as the corresponding metalloconjugates exhibited IC_50_ values within the range of 8 to 16 nM. ^68^Ga-PSMA I&T is characterized by rapid tumor targeting (4.95 ± 1.57 % IA/g at 1 h p.i.) and pharmacokinetics with high uptake in PSMA-positive organs such as the tumor and the kidneys (53.26 ± 9.02 % IA/g at 1 h p.i.). ^68^Ga-PSMA I&T is currently under clinical investigation.

**Figure 15:**
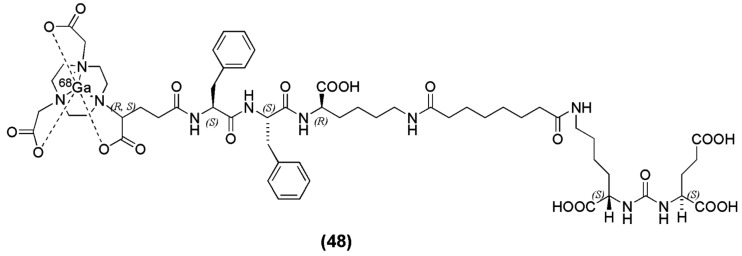
Chemical structure of ^68^Ga-(*R*)-NODAGA-Phe-Phe-d-Lys(suberoyl)-Lys-urea-Glu (^68^Ga-CC34) (**48**) [136].

The enantiomerically pure prochelator (*R*)-1-(1-carboxy-3-carbotertbutoxypropyl)-4,7-carbotartbutoxymethyl)-1,4,7-triazacyclononane ((*R*)-NODAGA(tBu)3) was reported by Gourni et al. for the functionalization of urea-based PSMA inhibitor derived from the Lys-urea-Glu peptidomimetic structure. The spacer Phe-Phe-d-Lys(suberoyl) was included to obtain (*R*)-NODAGA-Phe-Phe-d-Lys(suberoyl)-Lys-urea-Glu (CC34) (compound **48**, Figure 15) [136]. ^68^Ga-CC34 was obtained in high specific activity (75–80 MBq/nmol) and evaluated in vitro and in vivo in LNCaP tumor xenografts by biodistribution and PET imaging studies. ^68^Ga-PSMA-11 was also evaluated for comparison. ^68/natGa^-CC34 exhibited high affinity for the LNCaP cells, with a K_D_ value of 19.3 ± 2.5 nM. Tumor uptake of ^68^Ga-CC34 (14.5 ± 2.9 % IA/g) in LNCaP xenografts at 1 h p.i. was comparable to ^68^Ga-PSMA-11 (15.8 ± 1.4 % IA/g) (*p* = 0.67). The tumor-to-normal tissue ratios at 1 and 2 h p.i of ^68^Ga-CC34 were also comparable to that of ^68^Ga-PSMA-11 (*p* > 0.05).

**Figure 16:**
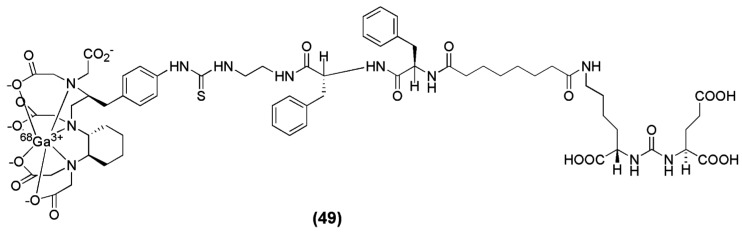
Chemical structure of ^68^Ga-CHX-A″-DTPA-DUPA-Pep (**49**) [137].

In another recent preclinical study, the cyclohexyl-diethylene triamine pentaacetic acid (CHX-A″-DTPA) was used as the chelator for the Glu-urea-GLu-based peptide (Pep) bearing a 2-[3-(1,3-dicarboxypropyl)-ureido]pentanedioic acid (DUPA) moiety to obtain CHX-A″-DTPA-DUPA-Pep (compound **49**, Figure 16). The study describes the efficient labeling of CHX-A″-DTPA-DUPA-Pep with ^68^Ga, ^90^Y and ^177^Lu and the first in vitro characterization which shows high affinity of the tested radiotracers towards PSMA with K_D_ values of ≤14.67 ± 1.95 nM. Labelling with ^68^Ga was performed at room temperature under neutral conditions (HEPES, pH 7.4). Significant differences in radiochemical yields were observed. Radio labelling with ^68^Ga succeeded in 10 min with high radiochemical yields; ≥95% when 50 μg (36 nM) of the peptide was used. However, in vivo images or biodistribution data of these agents have so far not been published [137].

### 4.3. ^64^Cu-Labeled Urea-Based PSMA Inhibitors

Amongst the PET-radioisotopes, ^64^Cu has gained particular attention because of its decay characteristics (t_1/2_ = 12.7 h; β^+^, Emax = 0.653 MeV [17.8%]; β^−^, Emax = 0.579 MeV [38.4%]) and the well-established coordination chemistry with a variety of chelators. The longer half-life of ^64^Cu relative to ^68^Ga (t_1/2_ = 67.8 min) and ^18^F (t_1/2_ = 109.8 min) is particularly attractive, as it allows PET-images at later time points with improved tumor-to-background ratios [138]. Additionally, ^64^Cu-labeled radiopharmaceuticals can be produced at a central facility and distributed to remote hospitals. The successful application of ^64^Cu as a diagnostic agent may also lead to potential targeted radionuclide therapy with ^67^Cu (t_1/2_ = 61.9 h; β^−^, E_max_ = 0.141 MeV [100%]) providing a promising theranostic pair.

**Figure 17:**
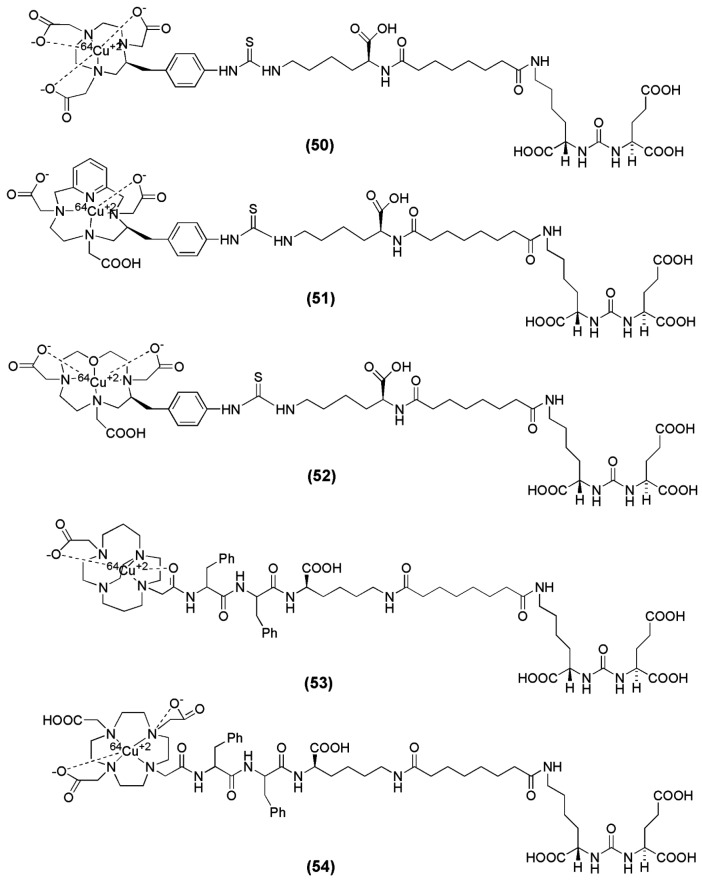
Chemical structures of the PSMA-based inhibitors (**50**–**54**) labeled with ^64^Cu [139].

Banerjee et al. reported the synthesis and preclinical evaluation of a series of five PSMA inhibitors labeled with ^64^Cu [139]. The main focus of the study was given on the effect of various chelators on PSMA-targeted ureas with respect to their pharmacokinetics for in vivo PET imaging. The investigated chelators are 1,4,7,10-tetraazacyclodoadecane-*N*,*N*′,*N*″,*N*′′′-tetraacetic acid, (DOTA), oxa-4,7,1-tetraazacyclododecane-4,7,10-triacetic acid, (Oxo-DO3A), 1,4,8,11-tetraazabicyclo[6.6.2]-hexadecane-4,11-diacetic acid, (CB-TE2A), 3,6,9,15-tetraazabicyclo[9.3.1]-pentadeca-1(15),11,13-triene)-3,6,9-triacetic acid (PCTA) and 1,4,7-triazacyclononane-1,4,7-triacetic acid (NOTA) (compounds **50**–**54**, Figure 17). The radiolabeling with ^64^Cu was performed in acetate buffer and the conditions differed based on the chelator (Table 6). The metal-free CB-TE2A-conjugated ligand **53** contained two nearly inseparable isomeric peaks by HPLC. This is most probably caused by the presence of an asymmetric center at the Phe moiety of the spacer, located very close to CB-TE2A. Radiolabeling of **53** resulted in two HPLC separable products, named ^64^Cu-53A and ^64^Cu-53B. All radioligands were obtained in specific activities in the range 2.9–9.1 GBq/μmol. ^64^Cu-53A, ^64^Cu-53B and ^64^Cu-54 were found to be more hydrophilic despite the presence of two Phe moieties at the spacer. The diastereomeric metalloconjuagtes 53A and 53B were the ones with highest affinity for PSMA. PET imaging as well as ex vivo biodistribution studies showed that ^64^Cu-50 and ^64^Cu-53A/B (NOTA and CB-TE2A chelators, respectively) exhibited high in vivo stability as evidenced by their lower liver uptake than that of the other three conjugates (blood: 1.06 ± 0.29, 1.87 ± 0.6, 0.20 ± 0.03 and 0.20 ± 0.07 % IA/g; liver: 8.63 ± 0.92, 17.04 ± 1.44, 1.68 ± 0.38 and 1.63 ± 0.72 % IA/g for ^64^Cu-50, ^64^Cu-51, ^64^Cu-53A and ^64^Cu-53B at 2 h p.i.). An additional finding of this study was that liver uptake and blood concentration were much lower at all time points for ^64^Cu-53A/B compared to that of ^64^Cu-50, suggesting a higher in vivo stability of CB-TE2A-conjugated ^64^Cu-53A/B than of NOTA-conjugated ^64^Cu-50. That might also be related to the higher hydrophilicity of ^64^Cu-53A/B compared to ^64^Cu-50. On the other hand, the high liver uptake and the slow blood clearance for the ^64^Cu-DOTA-conjugated radiotracer were indicative of free Cu(II), which is accumulated in liver [140,141]. Noteworthy is also the fact that negatively charged ^64^Cu-labeled NOTA-conjugated PSMA inhibitor, ^64^Cu-50, similarly to ^99m^Tc-oxo compounds [99], exhibited higher kidney and spleen uptake compared to ^64^Cu-53A/B (kidney: 125 ± 42, 26 ± 9 and 25 ± 11 % IA/g; spleen: 13.42 ± 1.18, 0.39 ± 0.30 and 0.90 ± 0.23 % IA/g for ^64^Cu-50, ^64^Cu-53A and ^64^Cu-53B at 2 h p.i.) Furthermore, although the PSMA-mediated renal uptake has been shown to be specific by several groups, it is interesting that variations are observed not only in regard to the absolute kidney uptake, but also with regard to the renal elimination.

The NODAGA-conjugated PSMA inhibitor CC34 (Figure 15), was also radiolabeled with ^64^Cu in ammonium acetate buffer, pH 5.4, to completion within 30 min at 95 °C, and was used without further purification. The specific activity of ^64^Cu-CC34 was 40 MBq/nmol and a log*P* of −3.01 ± 0.06. When CC34 was labeled with ^64^Cu, the resulting tracer exhibited a slightly higher lipophilicity than did ^68^Ga-CC34 (Log*P* = −3.54 ± 0.06) [136]. ^64^Cu-CC34 was preclinically evaluated in LNCaP xenografted mice. A high affinity towards PSMA on LNCaP cells was shown (K_D_ = 27.5 ± 2.7 nM). Furthermore, the versatility of NODAGA was exploited for efficient labeling also with ^64^Cu, which allows for the conduction of biodistribution/imaging studies at later time points compared to ^68^Ga-CC34, and thus a complete assessment of the pharmacokinetics of the radiotracer. ^64^Cu-CC34 is specifically taken up by the PSMA positive organs at early time points and washed out faster from the PSMA positive organs compared to uptake in tumor, leading to improved tumor to background ratios (tumor/blood: 41 ± 10 and 114 ± 17, tumor/muscles: 61 ± 18 and 103 ± 21 at 1 and 4 h p.i., respectively). The very low liver uptake of ^64^Cu-CC34 at all time points in combination with the short blood circulation, comparable to what was also reported for the CB-2A-conjugated PSMA inhibitor from Banerjee et al. [139] is a strong indication of the excellent in vivo stability of the ^64^Cu-NODAGA complex.

### 4.4. ^111^In-Labeled Urea-Based PSMA Inhibitors

^111^In has suitable nuclear characteristics (t_½_ = 2.8 d, E(γ) = 173, 245 keV) for use as an important SPECT label. Additionally, intraoperative gamma probes are now an important, well-established technology in the management of cancer, particularly in the detection of sentinel lymph nodes [140]. So far two already established PSMA inhibitors, DOTAGA-(I-y)fk(Sub-KuE) (also named PSMA-I&T) and PSMA-617, have been labeled with ^111^In, as companion nuclear probes for radioguided surgery and SPECT imaging [135,136,142].

^111^In-PSMA-I&T exhibited high PSMA-affinity (^nat^In-PSMA-I&T: IC_50_: 7.5 ± 1.5 nM) and enhanced internalization (104 ± 7%) compared to ^177^Lu-PSMA-I&T (76 ± 2%) into LNCaP cells. Biodistribution studies in LNCaP tumor-bearing mice (1 h p.i.) revealed slightly reduced background accumulation of ^111^In-PSMA-I&T compared to ^177^Lu-PSMA-I&T and comparable tumor uptake of both compounds. These findings led to a somehow improved tumor/background ratios for ^111^In-PSMAI&T compared to ^177^Lu-PSMA-I&T at 1 h p.i. (tumor/blood-, tumor/liver-, tumor/intestines-, and tumor/muscle-ratios of 34 ± 8, 32 ± 6, 53 ± 8, and 43 ± 6, respectively, versus 18 ± 9, 7 ± 3, 12 ± 3, and 14 ± 9 for ^177^Lu-PSMA-I&T) [135,142].

^111^In-PSMA-617 was prepared within 30 min at 95 °C in ammonium acetate buffer pH 5.4 and were used without any further purification with a specific activity of 10 MBq/nmol and a K_D_ value of approximately 5 nM The pharmacokinetics of ^111^In labeled naphthyl-containing DOTA-conjugated PSMA inhibitor, PSMA-617were excellent, with impressive tumor to background ratios over time (i.e., tumor/blood-, tumor/kidney-, and tumor-muscle ratios of 72 ± 19, 0.2 ± 0.01, 148 ± 61; 772 ± 153, 0.4 ± 0.04, 582 ± 24; 4768 ± 110, 9 ± 2, 2819 ± 864; 5343 ± 1033, 16 ± 2, 1987 ± 177 at 1-, 4-, 24-, and 48-h p.i. respectively) [136].

### 4.5. ^177^Lu-Labeled Urea-Based PSMA Inhibitors

The development of new radiolabeled specific biomarkers is mainly focused on the early diagnosis and prognostic prediction continuing with the adaptation of their therapeutic interventions that are individually optimized. Taking into account the theranostic approach, an ideal radiopharmaceutical should be able to combine the ability to be used for both diagnosis and therapy when labeled with a diagnostic or a suitable therapeutic radionuclide respectively. The beta emitter ^177^Lu can be considered as an appropriate therapeutic unit during the construction of a therapeutic nuclear probe due to its favorable characteristics (t_1/2_ = 6.73 days, E_βmax_ = 497 keV, E_γ_ = 113 keV (6.4%) and 208 keV (11%)).

The tailor-made DOTA-conjugated PSMA inhibitor, PSMA-617, has also been reported to be efficiently labeled with ^177^Lu [133] in addition to ^68^Ga. Radiolabeling with ^177^Lu reached a radiochemical yield of greater than 99% at low amount of precursor (0.5 mg, 0.5 nmol) in sodium acetate buffer, pH 5. The specific activity was in the range of 4–40 GBq/mmol. ^177^Lu-PSMA-617 exhibited high stability for at least 72 h, high binding affinity towards PSMA (K_i_ = 6.91 ± 1.32 nM for ^nat^Lu-PSMA-617) along with enhanced internalization rate into the LNCaP prostate cancer cells. ^68^Ga-labeled PSMA-617 was specifically internalized up to 17.67 ± 4.34% injected activity/10^6^ LNCaP cells and ^177^Lu-labeled PSMA-617 up to 17.51 ± 3.99% injected activity/10^6^ LNCaP cells, both at 37 °C. The in vivo performance of the ^177^Lu-PSMA-617 was characterized by high and retained tumor uptake combined with a rapid clearance from the kidneys within 24 h p.i., which led to improved tumor to background ratios over time (1058 (tumor to blood) and 529 (tumor to muscle), 24 h p.i.). It is worth noting that ^177^Lu-PSMA-617 exhibited similar pharmacokinetics as ^111^In-PSMA-617 [131]. ^177^Lu-PSMA-617 seems attractive for endoradiotherapy due to its higher tumor uptake at later time points, lower spleen accumulation, and the highly efficient clearance from the kidneys. The clinical evaluation of ^177^Lu-PSMA-617 is under way.

Under the same concept of the theranostic approach, the PSMA inhibitors DOTAGA-ffk(Sub-KuE) and DOTAGA-(I-y)fk(Sub-KuE) (also named PSMA I&T) were also labeled with ^177^Lu and evaluated preclinically [135]. Radiolabeling with ^177^Lu performed in ammonium acetate buffer, pH 5, and the radiolabeled products were achieved in a specific activity of more than 38 GBq/μmol. Both cold lutetium analogues exhibited high affinity towards PSMA (IC_50_: ^nat^Lu-DOTAGA-ffk(Sub-KuE): 13.1 ± 2.2 nM, ^nat^Lu-PSMA I&T: 7.9 ± 2.4 nM). Compared with ^177^Lu-DOTAGA-ffk(Sub-KuE), tumor targeting of ^177^Lu-PSMA I&T was fast, with the highest uptake in tumor (7.96 ± 1.76 % IA/g at 1 h p.i.) xenografts and kidneys (107 ± 16 % IA/g at 1 h p.i.) (both PSMA-specific). ^177^Lu-PSMA I&T exhibits suitable targeting and retention characteristics and an assessment of this compound for application in endoradiotherapy is currently ongoing.

## 5. Clinical Assessment of Radiolabeled PSMA Inhibitors: Current Status

### 5.1. SPECT Imaging with ^99m^Tc-Labeled PSMA Inhibitors

**Figure 18:**
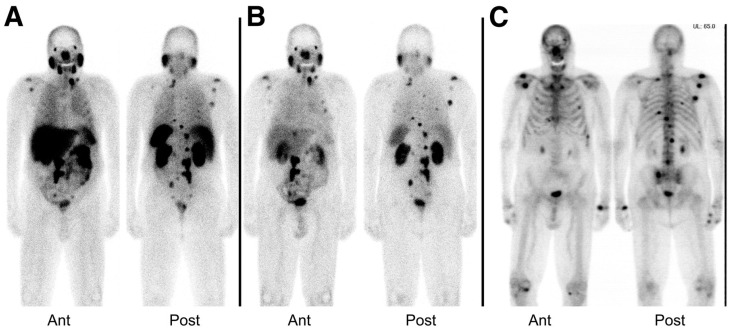
Tumor uptake of ^99m^Tc-MIP-1404 (**A**) or ^99m^Tc-MIP-1405 (**B**) at 4 h in patient with metastatic prostate cancer, compared with that of standard bone scan (**C**). Images also show uptake of radiotracer in normal parotid and salivary glands. Ant = anterior; Post = posterior (Reprinted with permission of [143]).

An exploratory investigational new drug application (IND) was implemented to bring the compounds MIP-1404 and MIP-1405 which been developed by the group at Molecular Insight Pharmaceuticals (MIP), and labeled with ^99m^Tc, into the clinic. Vallabhajosula et al. [143], reported the comparison of the pharmacokinetics, biodistribution, and tumor uptake of ^99m^Tc-MIP-1404 and ^99m^Tc-MIP-1405 in 6 healthy men and 6 men with radiographic evidence of metastatic prostate cancer. SPECT imaging was performed between 3 and 4 h after injection of the radiotracers (Figure 18). Both radiotracers showed fast blood clearance persistent uptake in the salivary, lacrimal, and parotid glands. They localized in bone and lymph node lesions as early as 1 h. Because of the lower urinary activity of ^99m^Tc-MIP-1404 (7%) compared to ^99m^Tc-MIP-1405 (26%), a clear advantage for detecting prostate cancer in the gland and pelvis at early stages of the disease is indicated for this compound. ^99m^Tc-MIP-1404 was selected for phase II studies to determine sensitivity and specificity to detect prostate cancer in high-risk patients. Furthermore, the development and optimization of a “cold” kit for the preparation of ^99m^Tc-MIP-1404 using generator eluted pertechnetate has also been reported [144].

### 5.2. PET Imaging with ^68^Ga-Labeled PSMA Inhibitors

**Figure 19:**
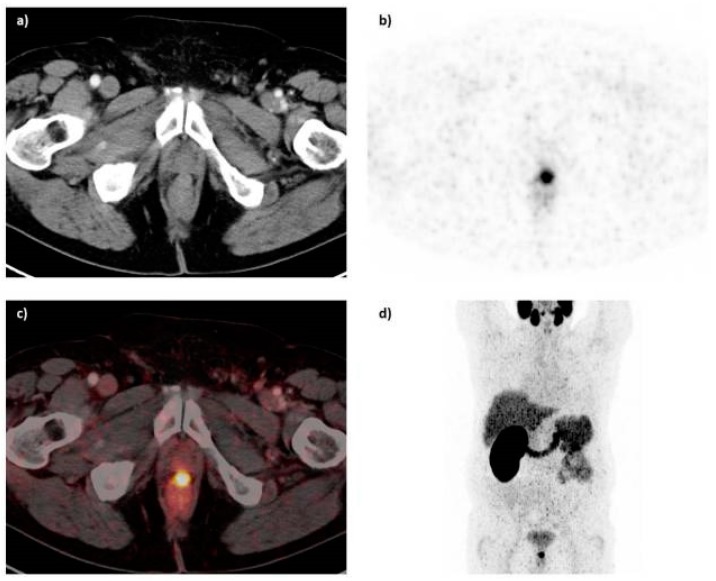
^68^Ga-PSMA-11 PET/CT image of a patient with locally recurrent prostate cancer (PSA 3.7 ng/mL) after radical prostatectomy (SUVmax 12.4) who received 140 MBq of the ^68^Ga-labeled tracer molecule and was scanned at 1 h p.i.; (**a**) CT image; (**b**) PET image; (**c**) PET/CT fusion image; (**d**) MIP (Reprinted with permission of [145]).

PET imaging of prostate cancer using PSMA-ligands has gained great attention during the last years. The numerous clinical trials evaluating ^68^Ga-PSMA-11 PET-CT imaging in prostate cancer patients have proved that this tracer can be used for lesion characterization, staging, planning and monitoring of therapy as well as evaluation of recurrence. ^68^Ga-PSMA-11 PET-CT imaging was firstly introduced in 2013 by Afshar-Oromieh et al. in 37 patients with relapsing prostate cancer (Figure 19) [107,145]. Within a relatively short time frame, ^68^Ga-PSMA-11 has been thoroughly clinically assessed by several research groups, indicative of the large number of publications the last four years [107,108,109,110,111,112,113,114,115,116,117,118,119,120,121,122,123,124,125,126,127,128,129]. Although the background uptake of ^68^Ga-PSMA-11 in salivary and lacrimal glands was abnormally high, PET-CT imaging was obtained leading to excellent detection of the metastatic lesions of prostate cancer.

Sterzing et al. [111] reported that PET-CT imaging with ^68^Ga-PSMA-11 may greatly contribute to the therapy planning of prostate cancer. The results of the study showed that ^68^Ga-PSMA-11 PET-CT had impact on the therapy regimen in 50.8% of the cases.

In a retrospective study, Afshar-Oromieh et al. [110] demonstrated that tumor detection mediated by ^68^Ga-PSMA-11 uptake was positively associated with prostate specific antigen (PSA) level and androgen deprivation therapy (ADT). Furthermore, ^68^Ga-PSMA-11 PET-CT imaging in patients with biochemical recurrence was proved to be crucial for recurrence evaluation, restaging as well as early treatment [112].

The ability of ^68^Ga-PSMA-11 to detect lesions of prostate cancer in patients with biochemical recurrence was also evaluated versus [^18^F]fluoromethylcholine [108,111]. In both studies ^68^Ga-PSMA-11 was found to be superior in detecting metastatic lesions associated with prostate cancer and displayed higher SUV_max_ values and superior tumor to background ratios. Another study by Dietlein et al. [113], PSMA-PET-CT imaging was conducting by applying ^68^Ga-PSMA-11 and ^18^F-DCFPyl in 14 patients with biochemical recurrence. ^18^F-DCFPyl was found to be somehow superior compared to ^68^Ga-PSMA-11 and could be considered as an alternative for PSMA-PET-CT imaging in relapsed prostate cancer.

**Figure 20:**
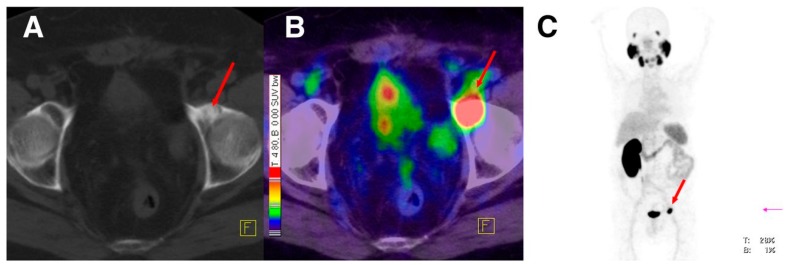
(**A**–**C**) ^68^Ga-PSMA-617 PET/CT of a patient at 1 h after injection. Red arrows point to a bone metastasis with SUVmax of 21.7 at 1 h and 32.6 at 3 h after injection. (**A**) Low dose CT; (**B**) Fusion of PET and CT; (**C**) MIP of PET/CT. MIP = maximum-intensity projection (Reprinted with permission of [146]).

The PSMA inhibitor PSMA-617 labeled with ^68^Ga was also clinically assessed in the diagnosis of prostate cancer by PET-CT [146]. ^68^Ga-PSMA-617 shows lesions of prostate cancer with high contrast, especially in late images (Figure 20). Maximum contrast of tumor lesions was achieved between 3 and 4 h after injection.

First-in-human ^68^Ga-PSMA I&T PET imaging allowed high-contrast detection of bone lesions, lymph node, and liver metastases [135]. In another study [143], ^68^Ga-PSMA I&T PET delayed images were also systematically evaluated after forced diuresis in 66 prostate cancer patients. The study explored the rather complex influence of delayed imaging and timing of forced diuresis on image quality. The authors suggest diuretic administration at 60 min p.i. in combination with delayed imaging at 180 min p.i. in order to obtain the maximum information possible, at least for ^68^Ga-PSMA I&T. However, the approach is time consuming [147].

### 5.3. PET Imaging with ^64^Cu-Labeled PSMA Inhibitors

The first clinical use of the ^64^Cu-labeled ligand PSMA-617 for PET imaging was conducted at two different centers in Austria and Germany [148]. 29 patients with either a high suspicion of recurrent disease were imaged in the context of therapy planning including surgery or PSMA radioligand therapy. The PET images showed an excellent resolution of the detected lesions with very high lesion to background contrast. An additional advantage of the ^64^Cu-labeled radiopharmaceuticals is the long half-life of ^64^Cu which allows distribution of the radiotracer to clinical PET centers where ^68^Ga-PSMA ligands are not available.

### 5.4. PSMA Radionuclide Therapy Using ^177^Lu-Labeled PSMA Inhibitors

**Figure 21:**
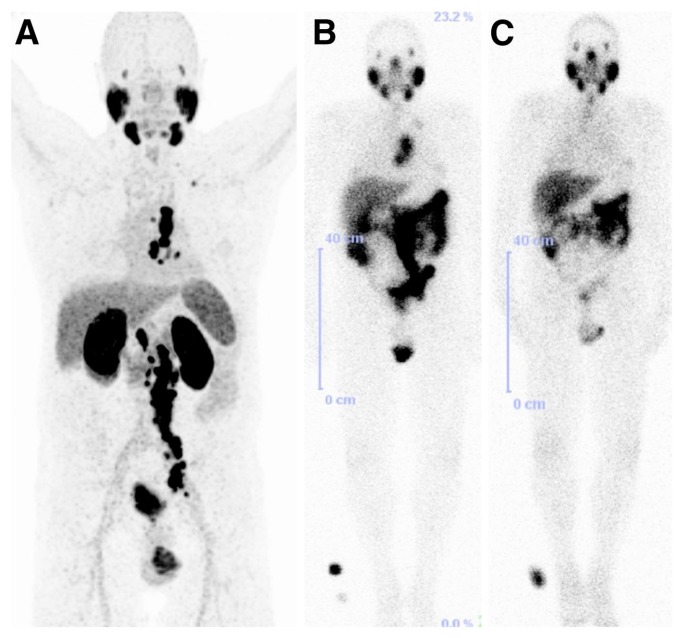
70-year-old patient with PSMA-avid lymph node metastases on ^68^Ga-PSMA PET/CT before therapy (**A**) and on ^177^Lu-PSMA I&T scintigraphy after first PSMA radionuclide therapy (**B**); with remarkable reduction in uptake after second PSMA RLT (**C**). Results were consistent with excellent therapy response projection (Reprinted with permission of [153]).

^177^Lu-labeled monoclonal antibody J591, which binds to the extracellular domain of PSMA, was firstly used for radionuclide therapy of prostate cancer [149]. Although the response to therapy was encouraging, the dose-limiting toxicity which is transient myelosuppression was the major reason of abandoning this therapeutic approach. In contrast, the development and preclinical evaluation of new PSMA ligands which could be labeled with therapeutic radionuclides such as ^177^Lu, paved the way for the reinvestigation of the ^177^Lu-based radionuclide therapy in patients with advanced prostate cancer. The radiotracers which have been used of the conduction of those studies are ^177^Lu-PSMA-617 and ^177^Lu-PSMA I&T (Figure 21). The clinical trials reported so far demonstrate promising results [150,151,152,153,154,155,156] with a mean tumor dose 6 to 12 fold higher compared to other critical organs such as kidneys and salivary glands. The PSA levels were found to be reduced in 60–90% of the patients after a single dose of both ^177^Lu-labeled PSMA-based radiotracers. The patients tolerated the therapy well, with no significant nephrotoxicity and just rarely occurring other severe side effects were observed. Although PSMA-directed radionuclide therapy bears a potential for implementation in routine clinical practice, the limited number of studies so far requires further controlled clinical trials for the establishment of the clinical value of ^177^Lu-PSMA-617 and ^177^Lu-PSMA I&T. ^177^Lu-PSMA-617 recently received FDA approval for phase II clinical trials in the U.S., (“First U.S. multi-center investigational clinical trial of ^177^Lu-PSMA-617 targeted radioligand therapy in metastatic castration resistant prostate cancer receives FDA clearance”. Daily Tribune, SyndiGate Media Inc. (Houston, TX, USA); 7 February, 2017, as found on http://www.pharmacychoice.com/News/article.cfm?Article_ID= 1690538).

### 5.5. Image-Guided Surgery of Prostate Cancer Lesions Using ^111^In-Labeled PSMA Inhibitors

The promising results of the diagnostic and endoradiotherapeutic approaches which have been successfully applied into the clinical setting using PSMA-based radiotracers rendered several research groups to make use of suitable PSMA-based radiotracers for other therapeutic strategies, such as the image-guided surgery of prostate cancer lesions. ^111^In-PSMA I&T ^111^In-PSMA-617 are the probes which have been used in two different clinical trials for radio-guided surgery for the detection of lymph node metastasis [117,142,157]. Both trials showed that all PET-positive lesions were also detected by PSMA-radio-guided surgery. Furthermore, tracer uptake and median gamma probe counts were significantly different between regions with lymph node metastases and other normal tissues. Overall, these studies showed that the gamma probes measurements could be considered as an indicator for lymph node metastasis and that radio-guided surgery may consequently lead to improvements with respect to the resection of prostate cancer.

## 6. Conclusions

Targeting the integral membrane glycoprotein PSMA by using radiometal-based PSMA inhibitors shows a great promise for diagnosis, (re)staging and therapy of prostate cancer. Specific PSMA-based ligands show high potential for initial staging, lymph node staging, restaging and therapy of prostate cancer. Between the different groups of PSMA inhibitors which have been developed so far, the peptidomimetic structure Lys-urea-Glu is the most successful biomolecule for utilization in radiopharmaceutical development. Furthermore, the choice of the chelator significantly affects the stability, affinity, lipophilicity and ultimately pharmacokinetics of the final radiolabeled PSMA inhibitor.

At the moment, ^99m^Tc-MIP-1404 seems to be the tracer of choice for SPECT imaging of prostate cancer, while ^68^Ga-PSMA-11 is one of the most promising tracers for PET imaging of patients with this disease. Due to the fact that ^68^Ga-PSMA-11 can be used only for diagnostic purposes, two modified PSMA inhibitors, PSMA-617 and PSMA I&T, have been developed. The superiority of PSMA-617 and PSMA I&T compared to PSMA-11 is that they can be efficiently labeled with both ^68^Ga for PET, as well as, ^177^Lu for radionuclide therapy. Therefore, both have the potential to be used as theranostic pairs. Although PSMA-directed radionuclide therapy shows great promise for implementation in routine clinical practice, the limited number of studies so far emphasizes the need for further controlled clinical trials for assessing the clinical value of ^177^Lu-PSMA-617 and ^177^Lu-PSMA I&T. Finally, image-guided surgery of prostate cancer lesions using ^111^In-labeled PSMA inhibitors seems to be an appealing approach for detection of lymph node metastasis.

## Figures and Tables

**Table 1:** Affinity, labeling and lipophilicity data of the ^99m^Tc-labeled PSMA inhibitors (**13**–**25**).

Compound	Inhibitory Activity (Ki (nM))	Labeling	Log*P*_oct/water_	Stage of Translation	Reference
**13–20**	0.03 (**13**)	0.25 M ammonium acetate/tartrate buffer, SnCl_2_ (4 mg/mL), ^99m^TcO_4_^−^, pH 8–8.5, 90–95 °C, 10–15 min, yield: >80%, purity: >99%, specific activity: >411 GBq/μmol	−2.38 (**13**)	no clinical translation	[99]
	0.13 (**14**)		−2.25 (**14**)		
	1.91 (**15**)		−2.14 (**15**)		
	7.99 (**16**)		−2.35 (**16**)		
	0.18 (**17**)		−3.04 (**17**)		
	0.048 (**18**)		N.D. (**18**)		
	0.17 (**19**)		−2.96 (**19**)		
	2.73 (**20**)		−2.94 (**20**)		
**21–24**	15.25 (**21**)	[Tc(CO)_3_(H_2_O)_3_]^+^, pH 7.2, 95 °C, 30 min, yield: 70–95%, purity: >98%, specific activity: >411 GBq/μmol	−2.03 (**21**)	no clinical translation	[98,99]
	1.12 (**22**)		−2.37 (**22**)		
	12.60 (**23**)		−2.05 (**23**)		
	16.30 (**24**)		−2.07 (**24**)		
**25**	1.74 (**25**)	HYNIC (2 mg/mL), 0.5 M NaHCO_3_, tricine (7 mg/mL), SnCl_2_ (1 mg/mL), ^99m^TcO4^−^, r.t., 30 min, yield: >80%, purity: >99%	−3.05 (**25**)	no clinical translation	[99]

**Table 2:** Affinity, labeling and lipophilicity data of the ^99m^Tc-labeled PSMA inhibitors.

Compound	K_D_ (nM)	Labeling	Log*P*_oct/water_	Stage of Translation	Reference
**26–31**	176 (**26**)	SnCl_2_, α-d-glucoheptonate, ^99m^TcO_4_^−^, pH 6.8, 90–100 °C, 18 min, yield: >98%, purity: >98%, Kit formulation was also performed	−3.94 (**26**)	no clinical translation	[100]
	60 (**27**)		−3.45 (**27**)		
	13.8 (**28**)		−0.27 (**28**)		
	31 (**29**)		−2.11 (**29**)		
	102 (**30**)		−4.64 (**30**)		
	338 (**31**)		−2.16 (**31**)		

**Table 3:** Affinity, labeling and lipophilicity data of ^99m^Tc-MAS_3_ (**32**)/mas_3_ (**33**)-y-nal-k-Sub-KuE.

Compound	IC_50_ (nM)	Labeling	Log*P*_oct/water_	Stage of Translation	Reference
**32** (MES_3_ chelator)	47.6 ± 2.5	SnCl_2_,ascorbic acid, tartrate, ammonium acetate, ^99m^TcO_4_^−^, pH 7.5–8, 90 °C, 20 min. Cartridge purification to remove colloidal ^99m^Tc species, specific activity: 25–37 GBq/μmol Kit formulation was also performed	−2.9		[101]
**33** (mes_3_ chelator)	39.7 ± 1.2		−3.0	Initial stage of clinical assessment	

**Table 4:** Affinity, labeling and lipophilicity data of ^99m^Tc-TMCE.

Compound	IC_50_ (nM)	Labeling	Stage of Translation	Reference
^99m^Tc-TMCE (**34**)	3.4	Tc(CO)_3_(H_2_O)_3_]^+^ (Isolink kit), pH 7.2, H_2_O, microwave, 110 °C, yield: 12–17%, purity: >98% (after HPLC purification)	no clinical translation	[102]

**Table 5:** Affinity, labeling and lipophilicity data of ^99m^Tc-MIP-1428 (**35**), ^99m^Tc-MIP-1405 (**36**), ^99m^Tc-MIP-1404 (**37**), ^99m^Tc-MIP-1427 (**38**).

Compound	K_D_ (nM)	Labeling	Stage of Translation	Reference
^99m^Tc-MIP-1428 (**35**)	1.75 ± 0.32	Tc(CO)_3_(H_2_O)_3_]^+^ (Isolink kit), pH 7.2, 100 °C, 30 min, purification via HPLC, yield: 70%, purity: >95%, specific activity: >37 TBq/mmol, Kit formulation was also performed	^99m^Tc-MIP-1404 is under clinical investigation	[103]
^99m^Tc-MIP-1405 (**36**)	4.35 ± 0.35			
^99m^Tc-MIP-1404 (**37**)	1.07 ± 0.89			
^99m^Tc-MIP-1427 (**38**)	0.64 ± 0.46			

**Table 6:** Affinity, labeling and lipophilicity data of the ^64^Cu-labeled PSMA inhibitors **50**–**54**.

Compound	K_i_ (nM) (^63/65^Cu-…)	Labeling	Yields (%)	Log*P*_oct/water_	Stage of Translation	Reference
^64^Cu-50	6.23	Acetate buffer, pH 5.5–6, 65 °C, 30 min	65–70	−1.17	no clinical translation	[139]
^64^Cu-51	10.76	Acetate buffer, pH 5.5–6, 65 °C, 30 min	70–90	−1.42		
^64^Cu-52	5.47	Acetate buffer, pH 5.5–6, 65 °C, 30 min	75–85	−1.56		
^64^Cu-53A	3.98	Acetate buffer, pH 7.5–8, 95 °C, 1 h	40–45	−2.68		
^64^Cu-53B	4.65		30–35	−2.31		
^64^Cu-54	13.26	Acetate buffer, pH 5.5–6, 65 °C, 30 min	65–70	ND		
